# Case-area targeted interventions and free chlorine residual in household drinking water: An observational cohort study during a cholera outbreak in Northeast Nigeria

**DOI:** 10.1371/journal.pntd.0012731

**Published:** 2025-01-27

**Authors:** Lindsay Salem-Bango, Jennifer OKeeffe, Michael R. Desjardins, Daniele Lantagne, Chiara Altare, Gurpreet Kaur, Kanaganathan Rangaiya, Patricia Oke-Oghene Obroh, Ahmadu Audu, Chimda Emmanuel Solomon, Thomas Heath, Emmanuel Emeka Ihemezue, Solomon Aye, Baptiste Lecuyot, Mustafa Sikder, Shannon Doocy, Melody Xiao, Paul B. Spiegel

**Affiliations:** 1 Center for Humanitarian Health, Department of International Health, Johns Hopkins Bloomberg School of Public Health, Baltimore, Maryland, United States of America; 2 Spatial Science for Public Health Center, Department of Epidemiology, Johns Hopkins Bloomberg School of Public Health, Baltimore, Maryland, United States of America; 3 Lancon Environmental, LLC, Somerville, Maryland, United States of America; 4 Action contre la Faim, Abuja, Nigeria; 5 Action contre la Faim, Paris, France; 6 Solidarités International, Maiduguri, Nigeria; 7 Solidarités International, Paris, France; Wayne State University, UNITED STATES OF AMERICA

## Abstract

**Background:**

Cholera outbreaks are surging worldwide. Growing research supports case-area targeted interventions (CATIs), whereby teams provide a package of interventions to case and neighboring households, as an effective strategy in cholera outbreak control, particularly in humanitarian settings. While research exists on individual CATI interventions, research gaps exist on outcomes of integrated interventions during CATI responses.

**Methodology/Principal findings:**

We conducted a prospective observational cohort study on CATIs during the 2021 cholera outbreak in Northeast Nigeria. During CATI response in Borno, Adamawa, and Yobe, research enumerators accompanied CATI teams to households and observed interventions (including provision of soap, Aquatabs, educational materials, and jerrycans; latrine and bedding disinfection; and hygiene promotion) and collected data on demographics, existing household water, sanitation, and hygiene, and household water free chlorine residual (FCR). Enumerators returned to households 10–14 days later to conduct follow-up surveys. We tested differences in reported delivery and receipt of interventions, and household drinking water FCR concentrations before and after CATIs. We also analyzed the associated relationship between CATI and environmental factors and odds of FCR <0.2 mg/L using quasi-Poisson multivariate logistic regression models with generalized estimating equations (GEE). We found household drinking water FCR significantly increased (p<0.001) post-CATI in Adamawa state. Self-reported receipt of Aquatabs and handwashing station availability were significantly associated with reduced odds of FCR <0.2 mg/L at follow-up. Self-reported receipt of hygiene promotion lacked significant associations with FCR in both Adamawa and Borno. These associations varied by type of water source.

**Conclusions/Significance:**

These findings suggest that CATIs improved household drinking water FCR, a key protective measure against cholera, in Northeast Nigeria. Our research highlights factors associated with FCR concentrations <0.2 mg/L post-CATI in Adamawa and Borno, offering valuable insights for response planning, and overall supports the continued use of CATIs in humanitarian settings.

## Author summary

Cholera, a bacterial disease impacting millions of people each year, is resurging worldwide. Case-area targeted interventions (CATIs) are an increasingly common cholera outbreak response strategy where teams deliver a mixture of health and water, sanitation, and hygiene (WASH) interventions to cholera case households and neighboring households within a specific radius. In particular, adding chlorine to water so that the concentration of free chlorine residual (FCR) is ≥0.2 mg/L can inactivate the bacteria that causes cholera. While studies have investigated the impacts of short-term WASH interventions including water treatment tablets and hygiene promotion, research is limited on integrated interventions during CATIs. We studied improvements in FCR concentrations following CATIs and factors associated with the odds of FCR concentrations <0.2 mg/L, collecting data during each CATI and following up with households 10–14 days later. We found significant improvements in household-level FCR concentrations following CATIs in Adamawa state. Factors associated with reduced odds of FCR levels <0.2 mg/L in Borno and Adamawa included self-reported receipt of water treatment tablets and handwashing station availability. Our findings support the continued use of CATIs in humanitarian settings and provide implementing partners with valuable insights for future CATI response.

## Introduction

Since 2021, the world has faced an upsurge of the seventh cholera pandemic, reversing decades of progress. Outbreaks have increased in number, size, geographic spread, and fatality. From 2021 to 2022, the number of countries reporting cholera cases rose by 25% (from 35 to 44)[1], and annual global case counts skyrocketed from 223,370 in 2021 to 472,697 in 2022.[1] Many countries have reported case-fatality rates (CFRs) higher than the maximum recommended threshold (CFR >1%); of the countries in the World Health Organization’s (WHO) African Region that reported cholera in 2022 and 2023, 65% reported CFRs >1% (range 1.1–12.3%).[2] Preliminary 2023 data suggests a continuation of these trends, with 667,000 cases and 4,000 deaths as of 15 December 2023, although the WHO cautions against direct comparisons at this time.[3]

Largely driven by climate change and political instability, this resurgence has particularly impacted fragile contexts facing multidimensional challenges like conflict, natural disasters, economic crises, and weakened health systems.[1] Experts are concerned that current trends will only continue as the climate and humanitarian emergencies grow.[4] Improved evidence-based interventions for cholera are urgently needed, particularly in conflict and fragile settings that pose specific challenges for response efforts.

In its Roadmap to reducing global cholera deaths by 90% by 2030, the Global Task Force on Cholera Control recommends more targeted interventions rather than blanket approaches.[5] One such targeted strategy is case-area targeted intervention (CATI). Supported by literature highlighting a person’s proximity to a cholera case as a key risk factor for cholera transmission [6–8], CATIs involve health (e.g., vaccination, case referrals) and water, sanitation, and hygiene (WASH) interventions (e.g., water treatment, hygiene kits, disinfection) at both the case household and neighbor households within a designated radius.[9] Growing research on CATI implementation supports its effectiveness in cholera outbreak control, with studies finding CATIs reduce case-cluster sizes, decrease infection among household contacts, and reduce overall cholera transmission.[10–13]

As more organizations and government ministries of health and water adopt CATIs, operational partners in low-resource settings must make critical decisions about what interventions to prioritize within CATIs. Growing research has sought to study the effectiveness of common cholera prevention measures; in particular, studies have investigated the impact of short-term WASH interventions including water treatment tablets (like Aquatabs) and hygiene promotion.[14,15] Water treatment tablets typically use chlorine to inactivate pathogens, improving drinking water quality. Sphere Standards outline that free chlorine residual (FCR) levels should be 0.2–0.5 mg/L to meet minimum water quality standards.[16] In cholera outbreaks, WHO generally recommends FCR concentrations ≥0.2 mg/L at the point of consumption and ≥0.5 mg/L at the point of distribution.[17] Hygiene promotion aims to improve knowledge and encourage behavior change of personal and household WASH practices to prevent disease transmission. Promotion topics can include treatment and use of safe drinking water, handwashing practice, food management, and use of improved sanitation facilities. Research has shown that both chlorine tablets and hygiene promotion can effectively improve water quality, although effectiveness varies widely across contexts.[18, 19] Additionally, the outcomes of these interventions have not been analyzed within an integrated CATI response. This is a critical gap, as CATIs currently incorporate many interacting interventions, some of which may be less effective than others, and target the highest risk households in a community (those living close to a case).

To address this gap, we studied improvements in household water FCR following CATIs and factors associated with concentrations <0.2 mg/L. The following inclusion criteria was used to select the areas/countries: 1) humanitarian setting or fragile state; 2) access to affected areas; and 3) occurrence of a cholera outbreak during the review time period. Decisions were made according to the results of the literature review and landscape analysis, and in the end Northeast Nigeria was chosen. We conducted a prospective observational research study in the conflict-affected states of Borno, Adamawa, and Yobe, Northeast Nigeria in 2021. That year, Nigeria experienced Africa’s largest cholera epidemic in 20 years, accounting for 50% of cases (n = 111,062) and 87% (n = 3,604, CFR = 3.2%) of cholera-related deaths globally. [1] The epidemic was concentrated in Northeast Nigeria, which has experienced prolonged insecurity that intensified the population’s vulnerability to cholera and hindered response efforts.[20] This study aims to add to the evidence for CATIs and identify associated factors that may influence CATI effectiveness.

## Methods

### Ethics statement

Solidarités International (SI) and Action contre la Faim (ACF) obtained Nigeria state-specific ethical approvals (SI: Borno SHREC Approval No. 050/2021; Yobe MOH/GEN/747/Vol. 1; Adamawa MENV/GEN/61/Vol.I/P.10, ACF: Borno SHREC Approval No. 052/2021; Yobe MOH/GEN/747/Vol. 1). Review from Johns Hopkins Bloomberg School of Public Health provided a determination of Public Health Surveillance (#14535) and non-research determination respectively. Adult participants provided informed verbal consent for participation, which enumerators documented.

### Study design and setting

We conducted a prospective observational cohort study to measure the effects of CATI response during the 2021 cholera outbreak in Northeast Nigeria. The study involved two “phases”: Phase 1 refers to the time during CATI implementation, while Phase 2 refers to the follow-up visits 10–14 days later. Two non-governmental organizations (NGOs), ACF and SI, conducted CATIs in the area participated in the study. The Borno response was concentrated in the state capital of Maiduguri, an urban city of 800,000 people including internally displaced persons. In Adamawa, the response centered on the peri-urban towns of Jimeta and Yola, home to 600,000 people. The Yobe response focused on the state capital of Damaturu, an agricultural region of 138,000 people.

The research presented herein was conducted as part of a larger mixed-methods study. Other quantitative results on the associated impact of CATIs on cholera clustering[10] and qualitative results on barriers to implementation[21] are published elsewhere. This manuscript focuses on household water FCR post-CATI to help identify factors that influence CATI effectiveness.

### CATI Design

The NGOs followed individual standard operating procedures (SOPs) for the initial CATI response. Upon receiving line lists from cholera treatment centers (CTCs), CATI teams should visit each case household and surrounding neighbor households within a 150-meter radius. At each household, teams were to conduct four key activities: (1) distribute supplies including Aquatabs, soap, and information, education, and communication (IEC) materials (two-month supply for case households and one-month supply for neighbor households, adjusted for household size); (2) conduct hygiene promotion; (3) disinfect key areas including latrines and bedding with chlorine solution; and (4) refer previously unidentified suspect cases to the CTC. Additionally, case households were to receive a jerrycan for water storage. Neither of the individual SOPs included oral cholera vaccine or antibiotic chemoprophylaxis.

### Data collection and management

Data collection occurred from September 15 to December 25, 2021. Duration varied by state: September 18-December 14 (87 days) in Adamawa, September 16-December 16 in Borno (91 days), and September 15-November 15 (61 days) in Yobe.

During CATI operations (Phase 1), enumerators embedded within the CATI teams used KoboToolbox [22] and mWater [23] data platforms to collect household-level data from case and neighbor households. Data covered household demographics, existing household WASH infrastructure (e.g., water source, handwashing station, latrine), observed interventions provided, and global positioning (GPS) coordinates (S1). Enumerators returned to households 10–14 days after the intervention to collect follow-up data (Phase 2), including demographics, household WASH infrastructure, GPS coordinates, household-report of interventions received during the initial CATI, and outcome indicators such as the FCR concentration of household drinking water (S1). Phase 2 household-level surveys were 33–48 questions long and a mix of enumerator observation and household member response. The survey was targeted towards any household member who was present, aged 18 years or older, and consented to participating in the survey.

Enumerators were trained to obtain consent, deliver questions without bias, and record answers. Informed consent was obtained before administering questions. During the survey, the respondent was asked, “Could you please provide me with a glass of water that members of your household usually drink?” Enumerators used Palintest visual comparators (pool testers) with diethyl paraphenylene diamine (DPD1) tabs to test provided water samples for FCR.

During data cleaning, households were excluded if GPS precision was >20m, case households were excluded if no neighbor households received a CATI visit, and neighbor households were excluded if they were located >300m from a case household (twice the SOP recommended radius). Additionally, households missing FCR concentration were dropped from analysis. In Adamawa, this included 0.2% in Phase 1 and 6% in Phase 2. In Borno, this included 0.9% in Phase 2. While Borno had less than 1% of FCR data missing in Phase 2 (and therefore was included in the Phase 2 regressions), 38.4% of FCR values were missing in Phase 1, causing the state to be excluded from the comparison of Phase 1 and Phase 2 FCR concentrations. Similarly, Yobe was excluded from the statistical analyses, as 51% of households in Yobe had missing Phase 2 FCR data; descriptive statistics on CATIs in Yobe can be found in S2 Appendix. Households were also dropped if they had a FCR concentration above 5.0 mg/L (<0.4% in Adamawa).

We used R version 4.2.1, [24] RStudio version 2022.07.2 + 576 [25] for all data management and analysis.

### Statistical analysis

Table 1 outlines the statistical analyses conducted on each state following aforementioned data cleaning and management. We present respondent, household, and intervention descriptive statistics as medians with interquartile range (IQR) for continuous variables, and totals with percentages for categorical variables. To assess the accuracy and discrepancies in CATI data, we compared follow-up household-report of interventions received with enumerator observation of interventions provided. We calculated an overall change in FCR concentrations pre- and post-CATI response. Data limitations prevented linking households from Phase 1 to Phase 2, thus Pearson’s Chi-squared tests were used to analyze statistical differences in household and team report of interventions and pre-and post-CATI FCR concentrations.

**Table 1:** Statistical Analyses by State.

Method	Adamawa	Borno	Yobe
Descriptive statistics	Included	Included	Included
Quasi-Poisson multivariate regressions of Phase 2 data	Included	Included	Excluded
Pearson’s Chi-square tests comparing Phase 1 and Phase 2 FCR data	Included	Excluded	Excluded

Descriptive statistics were conducted on all three states; descriptive statistics for Yobe can be found in S1. Multivariate regressions could not be conducted on Yobe as 51% of Phase 2 free chlorine residual (FCR) values were missing. Pearson’s Chi-Squared tests could only be conducted on Adamawa as Borno was missing 38.4% of FCR values from Phase 1.

Preliminary analyses indicated bias from missing data in certain covariates. To account for this, missing data were imputed using a random forest algorithm, generating 20 datasets. We then performed analyses on random forest imputed data sets, pooling results for odds ratios, confidence intervals, and p-values. To further ensure robustness, we employed multiple imputation by chained equations (MICE) at predictor correlations of 0.1, 0.2, and 0.3, generating 20 datasets per correlation level. Results from the random forest datasets were benchmarked against complete case data and MICE imputed data sets (see S3 Appendix).

To analyze the odds of FCR concentrations <0.2 mg/L at follow-up, we analyzed FCR data as a binary outcome variable (protective [≥0.2 mg/L] vs. non protective [<0.2 mg/L] FCR concentrations). We employed quasi-Poisson multivariate logistic regression models, using generalized estimating equations (GEE). The GEE approach in logistic regressions handled clustering in multi-level data at household and ring levels for FCR concentration, improving the robustness of standard error estimations and associated confidence intervals. Only neighbor households were included in the regression models, as neighbor households are an indicator of how interventions would affect transmission to households that had not yet had a cholera case.

Explanatory covariates in the regression models included a combination of intervention and follow-up variables (Table 2). Intervention covariates were continuous ring-level variables: distance from case to CTC in kilometers and ring coverage (percentage of households reached). Follow-up covariates were household-level. Binary variables included receipt of Aquatabs and hygiene promotion. Categorical variables were water source (piped, protected, or purchased/unimproved) and handwashing availability (soap with or without water, water only, none). Additional variables were excluded due to limited data availability or low prevalence in neighbor households. For example, jerry cans were distributed only to case households per SOPs and were observed in less than 1% of neighbor households. Inclusion of covariate and interaction terms was based on univariate and ANOVA comparisons and subject-matter expertise. They varied by model due to differences in implementation and ANOVA results by state.

We evaluated model fit using residual plots (see S4 Appendix). The residual plots were grouped by CATI ring to account for unmeasured confounding factors, insufficient dose-response relationships, or clustering effects. Residual plots assessed the relationship between predictor and the response variables, allowing for identification of overprediction, underprediction, or systematic biases. Additionally, ANOVA Wald tests were conducted to evaluate the significance of individual predictors and their contribution to the model.

**Table 2:** CATI and Environmental Covariates Used in Regression Models.

Variable	Type	Details
Aquatabs	Binary	Household reported receipt of Aquatabs
Hygiene Promotion	Binary	Household reported reception of hygiene promotion
CTC Distance	Continuous	Geographic distance between case household and closest CTC in kilometers; calculated with recorded GPS coordinates
Ring Coverage	Categorical	Proportion of households in 150m ring radius that received CATI; calculated by dividing the number of CATI households in the ring by the estimated total households in the ring ^a^
Water Source	Categorical	Type of water source used by the household: protected, piped, purchased, or unimproved ^b^
Handwashing Station	Categorical	Observed household handwashing station: Soap (with or without water), water only, or none

^a^ To estimate the number of households within each CATI ring, buildings were manually enumerated using ArcGIS Pro (version 2.9) [26] and Google Earth Pro. [27]

^b^ Protected water source: tube well/borehole, rainwater, public tap/standpipe, protected well, protected spring. Piped: piped into dwelling, piped to neighbor, piped to yard/plot. Purchased: bottled, cart with small tank, water kiosk, water sachet. Unimproved: tanker truck, unprotected well, surface water.

^c^ Most humanitarian organizations like SI and ACF use humanitarian WASH standards, such as the Sphere Standards [16] or UNHCR for WASH Access [28], rather than Sustainable Development Goal standards [29]. Thus, the terms improved and unimproved are used herein.

ACF: Action contre la Faim. CATI: case-area targeted intervention. CTC: cholera treatment center. SI: Solidarités International. WASH: water, sanitation, and hygiene.

## Results

### Household characteristics

During the CATI response in Borno and Adamawa, teams visited 1,591 case households and 36,297 neighbor households (Table 3). While teams in Borno followed up with more case households in Phase 2 than did teams in Adamawa (519 versus 334), the latter followed up with more neighbor households (5,327 versus 4,071) and a larger percentage of both case and neighbor households (74.1% versus 45.5% and 46.7% versus 16.4%, respectively).

**Table 3:** Respondent, Household, and Case-Area Targeted Intervention Characteristics.

	Adamawa		Borno	
**CATI Response**	**Case Households**	**Neighbor Households**	**Case Households**	**Neighbor Household**
Received CATI (Phase 1)	451	11,408	1,140	24,889
Revisited for Phase 2 Follow-Up	74.1 (334)	46.7 (5,327)	45.5 (519)	16.4 (4,071)
**Household Characteristics–Phase 2**				
Household Size ^a^	7 (5, 10)	5 (3, 7)	7 (5, 9)	5 (4, 7)
Respondent Age ^a^	34 (25, 45)	30 (23, 38)	35 (28, 45)	31 (25, 41)
Respondent Female ^b^	78.7 (263)	88.0 (4,688)	84.6 (439)	92.4 (3,761)
Highest Household Education Level ^b^				
*None*	21.7 (71)	13.7 (710)	86.9 (451)	87.1 (3,539)
*Islamic*	20.8 (68)	14.2 (735)	0.4 (2)	0.3 (14)
*Primary*	8.9 (29)	5.7 (296)	4.4 (23)	3.6 (145)
*Secondary*	37.0 (121)	45.7 (2,372)	5.8 (30)	6.1 (248)
*Post-secondary*	11.6 (38)	20.7 (1,076)	2.5 (13)	2.9 (116)
**Household WASH and Environmental Characteristics** ^**c**^ **–Phase 2**				
Water Source ^b^^,^ ^*d*^				
*Protected*	78.0 (227)	81.4 (3,721)	94.0 (455)	92.7 (3,586)
*Purchased*	12.0 (35)	9.6 (439)	3.7 (18)	5.2 (203)
*Piped*	7.6 (22)	8.3 (380)	2.3 (11)	2.0 (79)
*Unimproved*	2.4 (7)	0.7 (30)	0.0	0.0
Distance to CTC (km) ^a^	3.2 (1.5, 5.6)	-	5.5 (3.3, 7.8)	-
Time to Water Source (minutes) ^a^	5 (2, 10)	5 (3, 15)	20 (10, 30)	20 (10, 30)
Water Treatment ^b^				
*None*	64.7 (216)	60.5 (3,223)	46.5 (231)	78.2 (2,985)
*Add Bleach/Chlorine*	6.9 (23)	1.4 (76)	51.5 (256)	17.3 (660)
*Let Stand and Settle*	27.8 (93)	37.5 (2,000)	1.2 (6)	1.6 (61)
*Strain Through Cloth*	0.3 (1)	0.0 (0)	0.2 (1)	1.3 (48)
*Other*	0.3 (1)	0.5 (28)	0.6 (3)	1.6 (62)
Shared Latrine ^b^^,^ ^e^	66.5 (222)	72.2 (3,846)	83.4 (433)	82.6 (3,361)
Improved Latrine ^b^^,^ ^f^	9.9 (33)	9.6 (511)	4.8 (25)	4.7 (191)
Handwashing Availability ^b^				
*Basic (Soap +/- Water)*	52.1 (174)	53.7 (2,856)	40.2 (208)	19.9 (806)
*Limited (Water)*	23.7 (79)	22.5 (1,199)	48.2 (249)	68.5 (2,768)
*None*	24.3 (81)	23.8 (1,266)	11.6 (60)	11.6 (469)
Knowledge of Key Handwash Times ^b^				
*Before Food Preparation*	58.7 (196)	58.7 (3,129)	64.5 (335)	54.9 (2,235)
*Before Eating*	99.1 (331)	99.4 (5,295)	99.6 (517)	96.9 (3,944)
*After Using Toilet*	93.1 (311)	93.5 (4,982)	97.1 (504)	96.4 (3,926)
*Before Cleaning a Child*	35.0 (117)	29.9 (1,595)	49.1 (255)	46.0 (1,872)
*When Caring for a Sick Person*	13.8 (46)	11.7 (624)	30.6 (159)	20.2 (821)

^a^ (Median (IQR))

^b^ (% (n))

^c^ Most humanitarian organizations like SI and ACF use humanitarian WASH standards, such as the Sphere Standards [16] or UNHCR for WASH Access [28], rather than Sustainable Development Goal standards [29]. Thus, the terms improved and unimproved are used herein.

^d^ Protected water source: tube well/borehole, rainwater, public tap/standpipe, protected well, protected spring. Piped: piped into dwelling, piped to neighbor, piped to yard/plot. Purchased: bottled, cart with small tank, water kiosk, water sachet. Unimproved: tanker truck, unprotected well, surface water.

^e^ Shared latrine: Household shares latrine with at least one other household.

^f^ Improved latrine: flush, pour flush, pit latrine (with slab), composting toilet.

CATI: case-area targeted intervention. CTC: cholera treatment center. IQR: interquartile range. n: number. UNHCR: United Nations High Commissioner for Refugees. WASH: water, sanitation, and hygiene.

Households in Borno had poorer education and WASH characteristics compared to those in Adamawa, including a higher percentage with no formal education (86.9% [n = 451] of cases and 87.1% [n = 3,539] of neighbors), longer travel times to water sources (median 20 minutes [IQR 10,30] for both cases and neighbors), widespread latrine sharing (83.4% [n = 433] cases and 82.6% [n = 3,361] neighbors), and low availability of improved latrines (4.8% [n = 25] cases and 4.7% [n = 191] neighbors) and handwashing stations (40.2% [n = 208] cases and 19.9% [n = 806] neighbors). In both Borno and Adamawa, cholera case households were larger in size than neighbor households (median 7 [IQR 5.9,10] versus 5 [IQR 3,4.7]).

### Reporting of CATI Interventions

Reported receipt of CATI interventions differed by state, and particularly by household type (Table 4). In Adamawa, 53.6% (n = 179) of case households versus 5.3% (n = 281) of neighbor households reported receiving all intended supplies (Aquatabs, soap, IEC materials, and, if a case household, jerry can; referred to as “complete supplies”). Similarly, in Borno, 83.6% (n = 434) of case households versus 26.0% (n = 1,058) of neighbor households reported receiving complete supplies. This disparity continued with “complete activities” (receiving hygiene promotion, latrine disinfection, and bedding disinfection): 91.9% (n = 307) and 82.5% (n = 428) of case households in Adamawa and Borno, respectively, reported receiving complete activities, compared to only 24.7% (n = 1,315) and 61.7% (n = 816) of neighbor households. Examining receipt of all intended supplies and activities, 50.0% (n = 167) and 71.7% (n = 372) of case households in Adamawa and Borno, respectively, reported receiving both complete supplies and activities, compared to only 0.6% (n = 31) and 18.3% (n = 746) of neighbor households.

**Table 4:** Reported Case-Area Targeted Intervention Activities by Phase and State, Adamawa and Borno, Sept 15 to Dec 25, 2021.

	Adamawa			Borno		
	Household-Report (Phase 2)	Enumerator-Observation (Phase 1)	p-value	Household-Report (Phase 2)	Enumerator-Observation (Phase 1)	p-value
**Cases** (n)	334	403		519	881	
Supplies (% (n))						
*Aquatabs*	72.2 (241)	71.2 (287)	0.78	87.9 (456)	94.6 (833)	<0.001
*Soap*	54.8 (183)	57.1 (230)	0.53	92.3 (479)	93.1 (820)	<0.001
*Jerry Can*	53.6 (179)	56.8 (229)	0.38	86.1 (447)	94.0 (828)	<0.001
*Complete Supplies*^*1*^	53.6 (179)	56.8 (229)	0.38	83.6 (434)	92.6 (816)	<0.001
Activities (% (n))						
*Hygiene Promotion*	100.0 (334)	74.9 (302)	<0.001	93.1 (483)	99.4 (876)	0.021
*Latrine Disinfection*	98.8 (330)	99.8 (402)	0.042	98.3 (510)	99.3 (875)	<0.001
*Bedding Disinfection*	92.2 (308)	100.0 (403)	<0.001	88.8 (461)	99.3 (875)	<0.001
*Complete Activities*^*2*^	91.9 (307)	74.7 (301)	<0.001	82.5 (428)	98.3 (866)	<0.001
Complete CATI	50.0 (167)	54.8 (221)	0.18	71.7 (372)	91.4 (805)	<0.001
**Neighbors** (n)	5,327	10,927		4,071	21,726	
Supplies (% (n))						
*Aquatabs*	25.7 (1,370)	29.9 (3,272)	<0.001	27.8 (1,132)	50.9 (11,067)	<0.001
*Soap*	6.8 (360)	17.5 (1,907)	<0.001	47.6 (1,938)	35.4 (7,701)	<0.001
*Complete Supplies*^*1*^	5.3 (281)	17.4 (1,905)	<0.001	26.0 (1,058)	35.1 (7,619)	<0.001
Activities (% (n))						
*Hygiene Promotion*	98.6 (5,253)	65.4 (7,148)	<0.001	91.5 (3,724)	99.7 (21,660)	<0.001
*Latrine Disinfection*	97.5 (5,196)	99.9 (10,917)	<0.001	97.4 (3,966)	99.7 (21,670)	<0.001
*Bedding Disinfection*	24.9 (1,327)	99.9 (10,914)	<0.001	67.3 (2,739)	94.2 (20,472)	<0.001
*Complete Activities*^*2*^	24.7 (1,315)	65.3 (7,139)	<0.001	61.7 (2,514)	94.0 (20,417)	<0.001
Complete CATI	0.6 (31)	17.3 (1,889)	<0.001	18.3 (746)	34.8 (7,571)	<0.001

^1^ Complete Supplies means the household received all of the following supplies: Aquatabs, soap, IEC materials, and (if a case household) a jerry can.

^2^ Complete Activities means the household received all of the following activities: hygiene promotion, latrine disinfection, and bedding disinfection.

CATI: case-area targeted intervention. IEC: information, education, and communication. n: number.

Comparison of household reports in Phase 2 to enumerator observation in Phase 1 varied across states (Table 4). In Adamawa, a similar proportion of case households reported receiving all intended supplies and activities (“complete CATI”) as was observed by enumerators (50.0% vs. 54.8%, p>0.05). Among neighbor households, a significantly lower proportion reported receiving all intended supplies and activities than was observed during CATIs (0.6% vs. 17.3%, p<0.001). In Borno, significantly lower proportions of case and neighbor households reported complete CATIs than was observed during CATIs (83.6% vs. 92.6% for cases, p<0.001; 71.7% vs. 91.4% for neighbors, p<0.001). Comparisons for each individual supply and activity can be found in Table 4.

### Household Change in FCR concentrations

Before CATIs, all case households in Adamawa were below the recommended FCR threshold of ≥0.2 mg/L (Table 5). After CATIs, the percentage rose to 29.1% (n = 95) of surveyed case households. Neighbor households exhibited smaller though still significant changes, with an increase from 0.02% (n = 246) pre-CATI to 19.5% (n = 973) post-CATI.

**Table 5:** Improvements in Free Chlorine Residual Concentrations Among Cases and Neighbors Pre- and Post-Case Area Targeted Interventions, Adamawa.

	Adamawa		
	Pre-CATI	Post-CATI	p-value
**Cases** (n)	403	334	
≥0.2 mg/L, %(n)	0 (0)	29.1 (95)	
<0.2 mg/L, %(n)	100 (403)	70.9 (231)	<0.001
**Neighbor** (n)	10,927	5,327	
≥0.2 mg/L, %(n)	0.02 (2)	19.5 (973)	
<0.2 mg/L, %(n)	>99.9 (10,892)	80.5 (4,024)	<0.001

### CATI Intervention and Environmental Associations with Neighbor Household FCR concentrations

**Fig 1:**
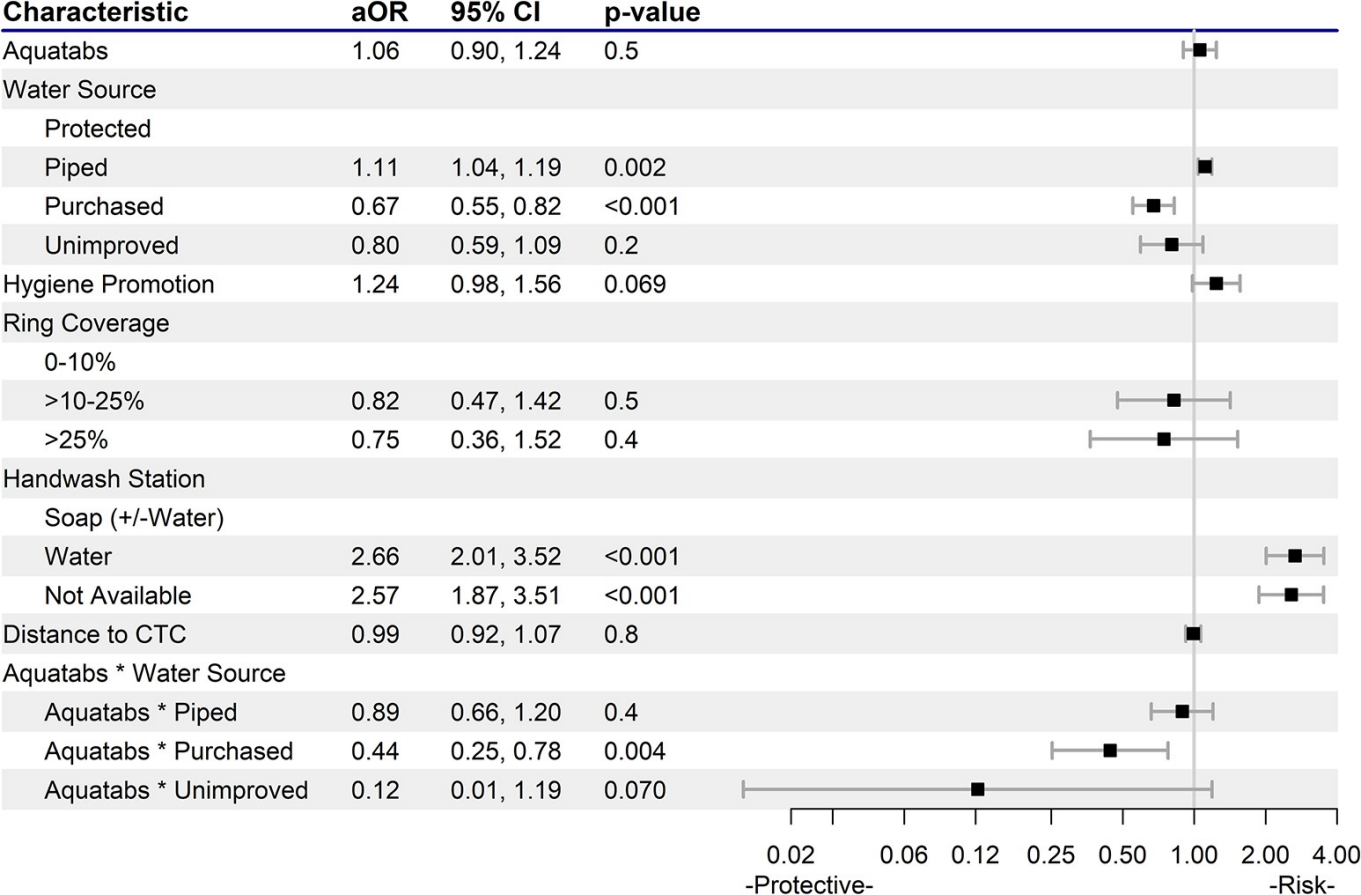
Adamawa State Adjusted Odds Ratios on Inadequate FCR Concentrations (<0.2 mg/L). Water source categories–Protected: tube well/borehole, rainwater, public tap/standpipe, protected well, protected spring. Piped water source: piped into dwelling, piped to neighbor, piped to yard/plot. Purchased: bottled, cart with small tank, water kiosk, water sachet. Unimproved: tanker truck, unprotected well, surface water. Ring coverage: The proportion of households in a ring that received a CATI visit. FCR: free chlorine residual; CTC: cholera treatment center.

**Fig 2:**
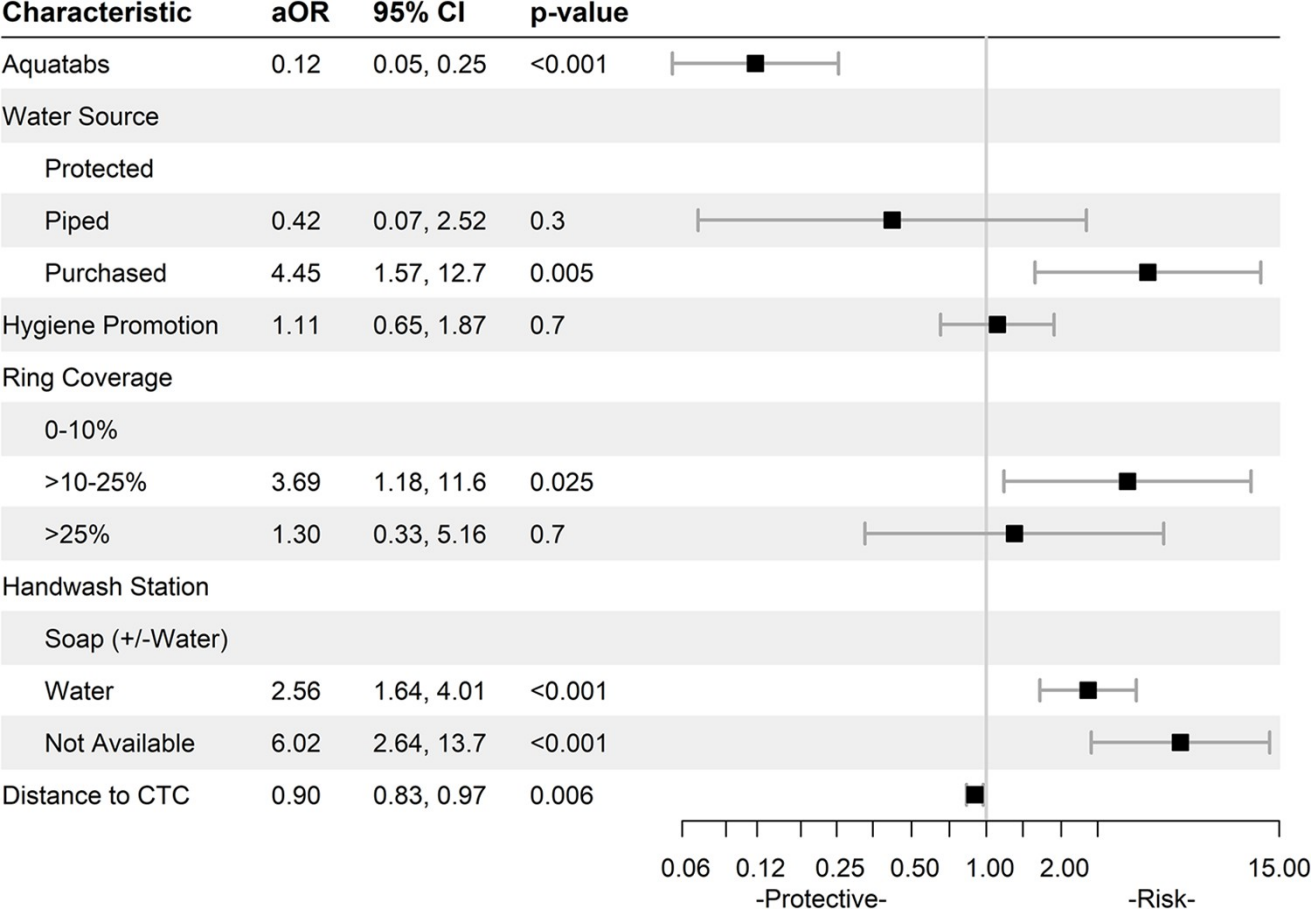
Borno State Adjusted Odds Ratios on Inadequate FCR Concentrations (<0.2 mg/L). Water source categories–Protected: tube well/borehole, rainwater, public tap/standpipe, protected well, protected spring. Piped water source: piped into dwelling, piped to neighbor, piped to yard/plot. Purchased: bottled, cart with small tank, water kiosk, water sachet. Unimproved: tanker truck, unprotected well, surface water. Ring coverage: The proportion of households in a ring that received a CATI visit. FCR: free chlorine residual; CTC: cholera treatment center.

The findings revealed varying impacts of CATI interventions, WASH, and environmental factors on FCR concentrations for neighbor households (Figs 1 and 2).

In Adamawa, the model demonstrated a reasonable fit, with most residuals distributed near zero and within acceptable bounds (S4 Appendix). Systematic overprediction was observed in rings with high FCR levels (≥0.2 mg/L). In Borno, model testing revealed underprediction in CATI rings with very low coverage (1–3%). These rings were excluded from the analysis as they did not provide a reliable representation of CATI, given the low dose. The final model in Borno showed good fit, with residuals within acceptable bounds.

In Adamawa, 4,997 observations were included in the analysis. The association of Aquatabs distribution with FCR concentrations varied by water source (Fig 1). For households using piped water, the distribution of Aquatabs did not enhance the odds of attaining high FCR concentrations compared to households using protected water sources. However, a significant protective effect was observed for households who received Aquatabs and used purchased water (aOR 0.44 [95%CI: 0.25, 0.78]; p = 0.004). For households using unimproved water sources, Aquatabs use showed a trend toward improved FCR (aOR 0.12 [95%CI: 0.01, 1.19]; p = 0.070), though the association was not significant and should be interpreted with caution due to the low number of observations. Without Aquatabs distribution, piped water showed greater odds of low FCR concentrations (aOR 1.11 [95%CI: 1.04, 1.19]; p = 0.002), and purchased water showed reduced odds (aOR 0.67 [95%CI: 0.55, 0.82]; p = <0.001) when compared to use of protected water sources.

In Borno, 3,979 observations were included in the analysis. Aquatabs distribution was associated with significantly lower FCR concentrations (aOR 0.12 [95%CI: 0.05, 0.25]; p <0.001) (Fig 2). Model testing showed this was consistent across water sources. Contrary to Adamawa, use of piped water revealed no differences in FCR concentrations compared to protected water sources, while use of purchased water showed increased odds of low FCR concentrations (aOR 4.45 [95%CI: 1.57, 12.7]; p = 0.005). In Borno, no households reported use of an unimproved water source.

In Adamawa, hygiene promotion approached, but did not show a significant association with low FCR concentrations (aOR 1.24 [95%CI: 0.98, 1.56]; p = 0.069). In Borno, hygiene promotion had no impact on household FCR concentrations. In Adamawa, ring coverage, defined by the percentage of households within a 150m radius visited during CATI responses, did not influence FCR concentrations, while in Borno, a ring coverage of 10% - 25% significantly increased the odds of inadequate FCR concentration in households (aOR 3.69 [95%CI: 1.18, 11.6]; p = 0.025).

In both states, the quality and presence of a handwashing station were strongly linked to household FCR concentrations. In Adamawa, households with a handwashing station with water, but no soap had greater odds of low FCR (aOR 2.66 [95%CI: 2.01, 3.52]; p<0.001), a pattern also seen in Borno (aOR 2.56 [95%CI: 1.64, 4.01]; p<0.001). Households without any handwashing station also exhibited greater odds of low FCR in both Adamawa (aOR 2.57 [95%CI: 1.87, 3.51]; p<0.001) and Borno (aOR 6.02 [95%CI: 2.64, 13.7]; p<0.001). Finally, while distance to the CTC had no impact in Adamawa, increased distance was protective against low FCR concentrations (aOR 0.90 [95%CI 0.83, 0.97]; p = 0.006) in Borno.

## Discussion

We studied FCR concentrations following CATI implementation during the 2021 cholera epidemic in the conflict-affected states of Borno and Adamawa in Northeast Nigeria. Our findings suggest that CATIs have the potential to improve FCR concentrations of household drinking water–a protective measure against cholera transmission. Our results also reveal disparities in outcomes highlighting opportunities to refine CATI to maximize impact and support response organizations and government ministries to make decisions on which interventions to prioritize.

In Adamawa, households that received CATIs experienced a significant increase in household FCR, indicating improved water quality. While contaminated water is not cholera’s sole transmission route, the significant improvement in FCR concentrations represents increased protection against transmission for at-risk households. However, most households still had FCR concentrations below the recommended 0.2mg/L, indicating room for improvement. Disparities between case and neighbor households were large, with case households more likely to achieve protective FCR concentrations. This disparity appears linked to differences in the quality of CATIs delivered. Both household-report (Phase 2) and enumerator observation (Phase 1) reported that fewer neighbors received key interventions, such as Aquatabs and hygiene promotion.

Aquatabs distribution among neighbors was strongly associated with protective FCR concentrations, aligning with previous findings that water treatment supplies improve FCR concentrations.[30] However, as in previous studies, outcomes varied by water source and state, highlighting the need for in-depth understanding of the outbreak context. Water quality can vary widely by source, even in areas that are geographically close.[31] In Borno, all households benefited from Aquatabs. In Adamawa, households using purchased water saw greater benefits than those with protected water sources.

Inconsistencies in FCR results by water source may reflect unmeasured factors, such as water availability and perceived water quality. Research in Tanzania found that fecal contamination and FCR concentrations were more influenced by water availability than type, with households experiencing water shortages less likely to have protective FCR concentrations. [32] Seemingly safe water sources might instill a false sense of security during cholera outbreaks. In Guinea-Bissau, ineffective pot-chlorinators in community wells led to reduced usage of water treatment tablets.[33] In this study, households may have been less inclined to treat water they perceived as safe, even though perceived quality may misalign with actual quality. [34] Cholera outbreaks have been linked to both unimproved and “improved” sources.[35–37]Additionally, studies highlight the degradation of FCR over time[38–40]; chlorinated municipal piped water may lose FCR before reaching households.[41]

These findings support the prioritization of water treatment tablets and effective risk communication within CATI packages. Ensuring sufficient supply remains a barrier to CATI effectiveness. In both Borno and Adamawa, most neighbor households reported not receiving Aquatabs. Qualitative interviews with response actors identified supply chain issues as a major operational challenge in CATI response.[21] Our research highlights the need for improvements in supply chain management across all levels of cholera preparedness and response, from individual CATI teams to organizational management to global agencies advocating for funding and supplies. Providing chlorine tablets to all households remains the optimal approach given the potential for rapid changes in water quality in fragile settings. However, uncontrollable supply shortages may require organizations to make difficult prioritizations. If supplies must be prioritized, organizations may consider focusing on households with low FCR concentrations. Furthermore, the provision of supplies alone is insufficient. Research in the Democratic Republic of the Congo, Kenya, Nepal, and Indonesia found many households had low FCR concentrations despite receiving water treatment tablets.[30,42] High-quality, community-driven health education with regular follow-up is critical to achieving greater usage of water treatment tablets. [43, 44] However, this requires sufficient staff, training, and resources. Time constraints limited CATI teams’ ability to conduct in-depth educational sessions.[21] In addition to improving staffing scale-up, implementing organizations must develop efficient, context-specific strategies to ensure proper tablet use after distribution.

The insignificant relationship between hygiene promotion and FCR concentrations in Borno and Adamawa likely reflects limited data variability, recall bias, and measurement bias. Few households reported not receiving hygiene promotion, and discrepancies existed between household and team reported hygiene promotion in both states. The study did not assess the quality of hygiene promotion, which were one-off occurrences. Given the strong evidence-base linking hygiene promotion to improved water quality, we included it in the final model. However, results should be interpreted alongside broader literature supporting hygiene promotion during cholera response.

Ring coverage showed limited influence on protective FCR concentration in both states. Higher ring coverage (>25%) was not associated with improved nor reduced FCR levels. One exception was observed in Borno, where 10–20% ring coverage was associated with low FCR concentrations, possibly due to unmeasured factors like urban or rural household location. Despite these findings, ring coverage remains a valuable metric for evaluating CATI reach and effectiveness. The accompanying impact study reinforces its role in managing cholera outbreaks. [10] Furthermore, ring coverage can serve an indicator of CATI quality, by providing a quantitative measure of the reach of CATI teams to targeted households.

Environmental factors also played a complex role in FCR outcomes, highlighting the challenges in using environmental factors to prioritize households during CATI response. In both states, the absence of handwashing stations with soap correlated with low FCR concentrations, pointing to broader environmental and hygiene conditions rather than the absence of soap itself. It may also reflect a lower quality CATI. As with Aquatabs distribution, few neighbor households received soap. Latrine sharing showed no correlation with FCR concentrations in either state, despite strong evidence linking it to cholera risk. [40] In Adamawa, distance to CTC was not linked to FCR concentrations, while in Borno greater distance to CTC was associated with higher FCR concentrations, likely due to unmeasured factors such as CTC location relative to outbreak hotspots.

This manuscript is part of a larger mixed-methods study on CATIs in humanitarian settings. The results presented herein provide quantitative support for challenges and recommendations identified in the qualitative component, including the need for increased staffing to improve CATI quality and stronger supply chain to prevent stock outs.[21] Our findings additionally reinforce the study’s quantitative results on the associated impact of CATIs on cholera clustering [10], identifying improved water quality as a likely mechanism for reduced clustering. Despite challenges highlighted in all components of the study, including high rates of incomplete CATIs, the study demonstrates that CATIs can still effectively combat cholera outbreaks.

This study has several limitations. First, selection bias was present as logistical constraints prevented follow-up to all households in a ring, especially in population-dense areas. Enumerators likely prioritized households closest to case households. Recall bias was also likely introduced, as follow-up occurred 10–14 days post-intervention, and household members present at the intervention may not have been present at follow-up. Furthermore, remote data collection due to the COVID-19 pandemic and security concerns reduced supervision, increasing the risk of measurement errors. Data collection occurred during an active cholera outbreak over a large coverage area, under significant resource and logistical constraints. As a result, key variables such as water storage practices and hygiene behaviors, which are known to influence water quality, were not systematically collected or included in the analyses due to data quality issues. These limitations may impact the interpretation of findings. Future research should investigate the role of these factors in household FCR concentrations and other CATI outcomes.

The absence of linked household identifiers between Phase 1 and Phase 2 precluded direct comparisons and prevented us from accounting for dependencies in the FCR pre- and post-CATI analyses. Although the study design planned for visits to both intervention and non-intervention households, follow-up data came predominantly from intervention households, preventing comparative analysis between the two groups. The analysis assumed all detectable FCR at follow-up originated from CATI-provided Aquatabs, while it may have come from other sources. Missing data in Yobe state precluded analysis, a notable gap given it had the largest follow-up sample. FCR is also one of many potential measures of CATI effectiveness. Data on acute watery diarrhea was collected but could not be analyzed due to poor data quality. Lastly, the study’s observational design restricts interpretation to associations of risk and protective factors rather than causal relationships.

These findings suggest that CATIs improved FCR concentrations in household drinking water, a critical factor in cholera prevention, during the 2021 cholera outbreak in Northeast Nigeria. They likewise highlight factors associated with reduced odds of FCR concentrations of <0.2mg/L, offering valuable insights for response planning. The findings support prioritizing Aquatabs in CATI response, strengthening supply chains to reach neighbor households, and delivering high-quality, community-driven education with regular follow-up. While FCR is just one measure of CATI effectiveness, this study builds on prior research linking CATIs to reduced cholera clustering. [10] Additional research is needed to identify the most influential CATI interventions to help organizations prioritize response activities in low-resource settings. Overall, the research supports the continued use of CATIs in fragile settings as a key tool in the global fight against cholera.

## Supporting information

S1 Appendix Phase 1 and Phase 2 Surveys. Source: OpenStreetMap (https://www.openstreetmap.org/#map=5/23.93/-102.57). (PDF)

S2 Appendix Descriptive Statistics, Yobe State. (PDF)

S3 Appendix Sensitivity Analyses. (PDF)

S4 Appendix Diagnostic and Model Fit Analyses. (PDF)
