# Supplementary material for: Case-area targeted interventions and free chlorine residual in household drinking water: An observational cohort study during a cholera outbreak in Northeast Nigeria
Source: PLoS Negl Trop Dis. 2025 Jan 27;19(1):e0012731. doi: 10.1371/journal.pntd.0012731 (PMC11771888; doi:10.1371/journal.pntd.0012731)
Supplement: S1 Appendix — (PDF) [file pntd.0012731.s001.pdf]

S1 Appendix: Phase 1 and Phase 2 Surveys

This appendix includes the following surveys:

1. Phase 1 surveys
    a. Action contre la Faim
        i. 1A Case Household
        ii. 1B Neighbor Household
        iii. 1C Ring
    b. Solidarités International
        i. 1A Household (both case and neighbor)
        ii. 1B Team (Ring)
2. Phase 2 surveys
    a. Action contre la Faim
        i. 2A Follow-Up Household
        ii. 2B Follow-up Team (Ring)
    b. Solidarités International
        i. 2A Follow-Up Household
        ii. 2B Follow-Up Team (Ring)

1A. Cholera - CATI - Case Household Monitoring

Household Information

Name of the staff members or RTT Member ?

Action Against Hunger is evaluating the effectiveness of its Cholera Response. To do this we are collecting the GPS coordinate of the locations we are working with our research partner. If you accept we will share this location, but all information will be anonymous and confidential. If you refuse, we will complete our intervention but not record any data. The project aims to understand how we can improve responses to cholera. Do you consent?

Hint: If the household does not consent, still complete the form but note we will not share the data

- ◯ Yes
- ◯ No

Record GPS current location of Cholera Household

Comments...

Which State are you in?

- ◯ Kano
- ◯ Buach
- ◯ Other (please specify)

What is the name of the community where the CATI was completed

What is the address of the household

Describe how to find the house

Location type:

◯ Urban - formal neighborhood
◯ Peri-urban - informal neighborhood
◯ Rural or dispersed population
◯ Refugee/IDP camp

Type of household

◯ Single house
◯ Apartment
◯ Attached

What is the Patient unique ID

Hint: Corresponding to the ID used in the Cholera Treatment Facility registration form and ACF intervention monitoring database

☐ Don't Know

Patient Name and Surname

Which CTC did the Cholera Patient Visit? OLD

☐ Don't Know

Which CTC did the Cholera Patient Visit?

◯ Specialist Teaching Hospital Bauchi
◯ General hospital Sumaila-Kano
◯ Sitti PHC-Kano
◯ Gani PHC-Kano
◯ PHC gomo-Kano
◯ Massu PHC-Kano
◯ Magami PHC- Kano
◯ Rimi PHC-Kano
◯ Other (please specify)

☐ Don't Know

CTC admission date (what date did the patient go to the CTC)

☐ Don't Know

Household Intervention Checklist

What is the Chlorine Free Chlorine Residual of the households water storage? (

Hint: Use DPD 1 to test the FCR with a pool tester to tes the water before you treat it

Select all items that are distributed to the household

☐ Chlorine tablet or solution to disinfect water
☐ Jerry can or water storage container
☐ Soap and/or laundry powder
☐ IEC materials

Select all activities that are completed at the household

☐ HH/bedding disinfection
☐ Latrine disinfection
☐ Hygiene promotion session
☐ HH water tank/storage chlorination
☐ Free Chlorine Residual of water testing (before treatment)
☐ Case referral
☐ Case Investigation

Observe: The type of toilet facility used by the household?

Hint:

◯ Flush / pour flush
◯ Bucket
◯ Pit latrine: Pit latrine with slab
◯ Pit latrine: Pit latrine without slab / Open pit
◯ Pit latrine: Composting toilet
◯ Hanging toilet
◯ No facility / bush / field
◯ Other (please specify)

Observe Type of hand washing facility

◯ Water and soap
◯ Water and other cleaning product
◯ Only water
◯ No handwashing facility
◯ Could not observe the handwashing facility

Select the main water source used by the household?

- ◯ Piped into dwelling
- ◯ Piped to yard / plot
- ◯ Piped to neighbor
- ◯ Public tap / standpipe
- ◯ Tube well / borehole
- ◯ Protected well

[ ]

- ◯ Unprotected well
- ◯ Protected spring
- ◯ Unprotected spring
- ◯ Rainwater
- ◯ Tanker-truck
- ◯ Cart with small tank
- ◯ Water kiosk
- ◯ Surface water (river, dam, lake, Pond, stream, canal, irrigation channel)
- ◯ Bottled water
- ◯ Sachet water
- ◯ Other (please specify)

[ ]

Is the water point within 500m

- ◯ Yes
- ◯ No
- ◯ Dont Know

If Is the water point within 500m is Yes:

Record the GPS location of the water point

[ ]

If Is the water point within 500m is Yes:

Take a photo of the water point

[ ]

If Is the water point within 500m is Yes:

Did you chlorinate the the water point ?

- ◯ Yes
- ◯ No
- ◯ Other (please specify)

[ ]

If Is the water point within 500m is Yes:

Any comments on the water chlorination

○ End Survey

1B Cholera –CATI Neighbours Household Monitoring

General

Enumerator's name:

What is the Patient unique ID of the Case Household for this CATI Ring

Hint:

What is Name of the Cati Household for this Ring

Date:

Case Information

Action Against Hunger is evaluating the effectiveness of its Cholera Response. To do this we are collecting the GPS coordinate of the locations we are working with our research partner. If you accept we will share this location, but all information will be anonymous and confidential. If you refuse, we will complete our intervention but not record any data. The project aims to understand how we can improve responses to cholera. Do you consent?

Hint: If the household does not consent, still complete the form but note we will not share the data

◯ Yes
◯ No

GPS:

Hint: GPS of the household

What is the name of the community where the CATI was completed

Type of household:

◯ Single house
◯ Apartment
◯ Attached

Household Checklist

Select all items that are distributed to the household

☐ Chorine tablet or solution to disinfect water
☐ Jerry can or water storage container
☐ Soap and/or laundry powder
☐
☐ IEC materials
☐ Other (please specify)

Select all activities that are completed at the household

☐ HH/bedding disinfection
☐ Latrine disinfection
☐ Hygiene promotion session
☐ HH water tank chlorination
☐ Water testing for FCR
☐ Case investigation
☐ Case referral

If Select all activities that are completed at the household includes Water testing for FCR:

FCR reading (in mg/L)

Select the main water source used by the household:

◯ Piped into dwelling
◯ Piped to yard/plot
◯ Piped to neighbor
◯ Public tap/ standpipe
◯ Tube well/ borehole
◯ Protected well
◯ Unprotected well
◯ Protected spring
◯ Unprotected spring
◯ Rainwater
◯ Tanker-truck
◯ Cart with small tank
◯ Water kiosk
◯ Surface water (river, dam, lake, pond, stream, canal, irrigation channel)
◯ Bottled water
◯ Sachet water
◯ Other (please specify)

Select the type of toilet facility used by the household:

◯ Flush/ pour flush
◯ Pit latrine
◯ Open pit
◯ Composting toilet
◯ Bucket
◯ Hanging toilet
◯ No facility/ bush/ field
◯ Other (please specify)

Select the type of handwashing facility:

◯ Water and soap
◯ Water and other cleaning product
◯ Only water
◯ No handwashing facility
◯ Could not observe the handwashing facility

End Survey

◯ End

1C Post Cholera CATI Completion Checklist

Untitled Section

This survey is to be completed after the CATI team has finished the intervention

Name of RRT Team Member

What State are you in

◯ Bauchi
◯ Kano
◯ Other (please specify)

Which CTC did the Cholera Patient Visit?

☐ Don't Know

What is the name of the community where CATI was completed

How long was the team at the site (in minutes)

What are the names of the people in the CATI team?

How many people in the CATI team?

Team members' skills/backgrounds

☐ WASH
☐ Nurse/Health
☐ Sprayer
☐ Surveillance
☐ Other (please specify)

What was the neighbor selection process used

◯ Neighbors from all sides of the case HH using specific radius
◯ A specific number of neighbors around the case HH
◯ Neighbors only along the case HH street
◯ Neighbors on the same apartment floor as case HH
◯ Neighbors that share same water source as case HH
◯ Other (please specify)

If What was the neighbor selection process used is Neighbors from all sides of the case HH using specific radius:
What is the radius/distance used to include neighbors (in meters)

How many households were initially planned to visit (case + neighbors)?

How many households were ultimately included in the CATI (case + neighbors)?

How many households were not included because no one was at the house?

How many households were not included because they declined?

How many households were not included for any other reasons?

Were there any challenges faced in delivering the CATI?

◯ End survey

Phase 1A Observation Household Form 8.10.21

General

Unique Household ID: 1117000000000

Observation CATI Form Number:

Please write the "Observation CATI Form" number here

Enumerator's name

Date

yyyy-mm-dd

Case Information

Is it a case or neighbor household?

- ◯ Case
- ◯ Neighbor

State:

- ◯ Adamawa
- ◯ Borno
- ◯ Yobe

LGA:

Ward:

Community/neighborhood:

Street address:

GPS
GPS of the household

latitude (x.y °)

longitude (x.y °)

altitude (m)

accuracy (m)

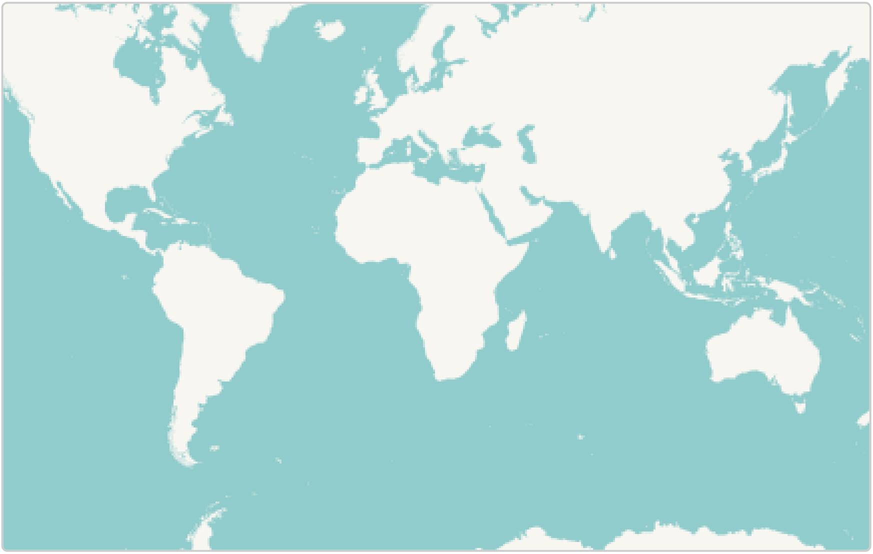

Source: OpenStreetMap (https://www.openstreetmap.org/#map=5/23.93/-102.57)

Type of household	Yes	No
Single house	○	○
Apartment	○	○
Attached	○	○

Household Checklist

Select "Yes" for all items that are distributed to the household	Yes	No
Chlorine tablet or solution to disinfect water	○	○
Jerry can or water storage container	○	○
Soap and/or laundry powder	○	○
ORS	○	○
IEC materials	○	○
Other	○	○

Other

Select "Yes" for all activities that are completed at the household	Yes	No
HH/bedding disinfection	○	○
Latrine disinfection	○	○

**Hygiene promotion session**	○	○
**HH water tank chlorination**	○	○
**Water sample collection**	○	○
**Water testing for FCR**	○	○
**Case investigation**	○	○
**Chemoprophylaxis administration**	○	○
**OCV administration**	○	○
**Case referral**	○	○

FCR reading (in mg/L):

____

Select the main water source used by the household?

- ○ Piped into dwelling
- ○ Piped to yard / plot
- ○ Piped to neighbor
- ○ Public tap / standpipe
- ○ Tube well / borehole
- ○ Protected well
- ○ Unprotected well
- ○ Protected spring
- ○ Unprotected spring
- ○ Rainwater
- ○ Tanker-truck
- ○ Cart with small tank
- ○ Water kiosk
- ○ Surface water (river, dam, lake, Pond, stream, canal, irrigation channel)
- ○ Bottled water
- ○ Sachet water
- ○ Other (specify)

Other

____

Select the type of toilet facility used by the household?

- ◯ Flush / pour flush
- ◯ Pit latrine
- ◯ Open pit
- ◯ Composting toilet
- ◯ Bucket
- ◯ Hanging toilet /
- ◯ No facility / bush / field
- ◯ Other

other

____

Select the type of handwashing facility

- ◯ Water and soap
- ◯ Water and other cleaning product
- ◯ Only water
- ◯ No handwashing facility
- ◯ Could not observe the handwashing facility

Phase 1B Observation CATI Team Form 8.10.21 v2

General

Observation CATI Form: 1117000000000

Enumerator's name:

Date

yyyy-mm-dd

CTC Information

Corresponding CTC:

- ◯ CTC1
- ◯ CTC2
- ◯ CTC3

CTC case ID: (enter the unique case ID from line list)

CTC admission date

yyyy-mm-dd

Case status:

- ◯ Suspected
- ◯ RDT positive
- ◯ Culture positive

CATI Team Information

CATI Team Number

The unique identifying number given to the team by the implementing organization.

Number of members in the team:

**Team member's skills/background:**	Yes	No
**WASH**	○	○
**Nurse/Health**	○	○
**Sprayer**	○	○
**Surveillance**	○	○
**Other**	○	○

Other:

Case household

State:

- ○ Adamawa
- ○ Borno
- ○ Yobe

LGA:

Ward:

Community/neighborhood:

Street address:

Location type:

- ○ Urban - formal neighborhood
- ○ Peri-urban - informal neighborhood
- ○ Rural or dispersed population
- ○ Refugee/IDP camp

Case Household's GPS

latitude (x.y °)

longitude (x.y °)

altitude (m)

accuracy (m)

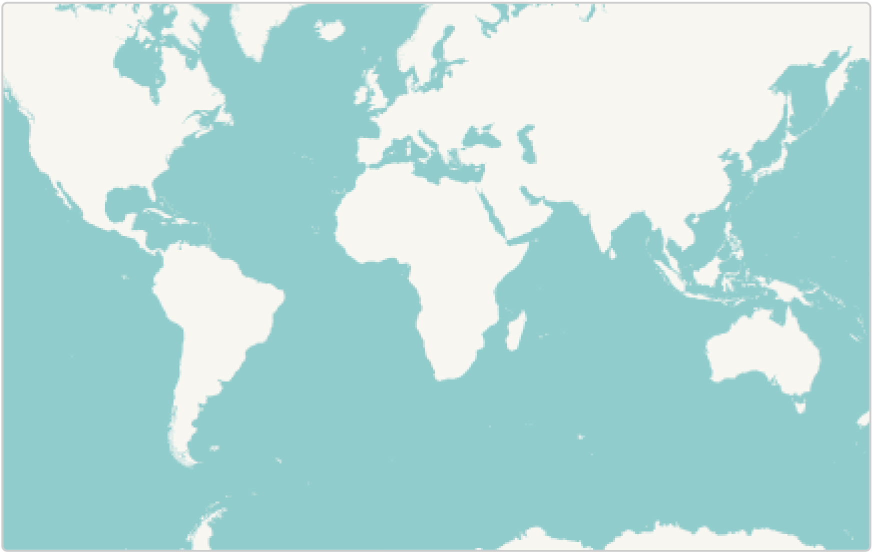

Source: OpenStreetMap (https://www.openstreetmap.org/#map=5/23.93/-102.57)

CATI Ring (complete this section when the CATI team completes intervention)

Neighbor selection process:

- ◯ Neighbors from all sides of the case HH using specific radius
- ◯ A specific number of neighbors around the case HH
- ◯ Neighbors only along the case HH street
- ◯ Neighbors on the same apartment floor as case HH
- ◯ Neighbors that share same water source as case HH
- ◯ Other selection process

Explain other selection process:

What is the radius/distance used to include neighbors (in meters)

How many households were initially planned to visit (case + neighbors)?

How many households were ultimately included in the CATI (case + neighbors)?

How many households were not included because no one was at the house?

____

How many households were not included because they declined?

____

How many households were not included for any other reasons?

____

Please write the other reason for not including the households:

____

Were all items (e.g. chlorine tablet/solution, water container, ORS, etc.) distributed to the case and neighbors' household according to the guideline/SoP?

- ◯ Yes
- ◯ No

If no, which items were not distributed according to the guideline/SoP and why?

____

Were there any challenges faced in delivering the CATI?

____

Phase 2A: Follow Up CATI HH Form D&D revised

General

Follow up CATI Form number:
Hint: Please write the "Follow up CATI Form" number here.

State

- ◯ Yobe
- ◯ Borno
- ◯ Other (please specify)

LGA

Name of the Community

Enumerator's Name

Is it a case or neighbor's household?

- ◯ Case
- ◯ Neighbor

Consent and Respondent's Information

WE WILL ONLY SURVEY RESPONDENTS WHO ARE 18 YEARS OR OLDER. Please ask the respondent about their age. If the respondent is younger than 18 years, please request to interview someone from the household who is over 18 years. Please read the follow-up survey consent form to the respondent and obtain oral consent to conduct the survey.

Hello. I am working with Johns Hopkins University and Action Against Hunger and would like to talk to you about a study on cholera reduction in humanitarian settings.

We are interested in learning about the effectiveness of interventions for reducing new cholera cases during outbreaks. I would like to ask you to join our study because you live in a community where cholera cases recently occurred. We want to learn if your household received the intervention and if any of your household members have recently had diarrhea. You do not have to participate; it is your choice. There will be no penalty if you decide not to join.

If you agree, we will conduct a brief interview. The interview will include questions on cholera control interventions, whether any household member has recently had diarrhea, your knowledge of cholera, and your household's hygiene behaviors. I will also collect a sample of your drinking water for testing. We expect this will take approximately 30 - 45 minutes. You may decide not to respond to a particular question if it makes you uncomfortable and you may also stop the interview at any time.

If you participate, there is a small risk someone outside the study will see your information. We will do our best to keep your information safe. The study findings will be reported for all participants combined so that no individuals can be identified. If data is shared with other researchers, we will make sure that no information that can identify you or your household is included.

We will not pay you and there is no direct benefit to participating in the study. We will use the interview information and the result of your drinking water test to evaluate if the interventions were effective in preventing the spread of cholera. . We hope this knowledge will improve future cholera response efforts.

Do you have any questions? You may contact [local partner name and contact info]about your questions or problems with this work. You may also contact the Johns Hopkins School of Public Health Institutional Review Board (Tel. 1-888-262-3242, jhsph.irboffice@jhu.edu) which approved this study, if you have questions about your rights as a study participant, if you feel you have not been treated fairly, or if you have other concerns.

Do you agree to participate?

- ◯ Yes
- ◯ No
- ◯ No adult at the household

Respondent is:

- ◯ Female
- ◯ Male

Age of the respondent is:

Take GPS location

[ ]

If Do you agree to participate? is Yes:

In the past month, did your household receive a visit from a CATI team?

- ◯ Yes
- ◯ No

If Do you agree to participate? is Yes and In the past month, did your household receive a visit from a CATI team? is No:

Why did you not receive CATI intervention?

- ◯ The CATI team did not come to the house
- ◯ We did not want the CATI team to come to the house
- ◯ No one was at the house when the CATI team came
- ◯ Do not know
- ◯ Other (please specify) (https://www.openstreetmap.org/#map=5/23.93/-102.57)

[ ]

If Do you agree to participate? is Yes and In the past month, did your household receive a visit from a CATI team? is No and Why did you not receive CATI intervention? is We did not want the CATI team to come to the house:

You said you did not want the CATI team to come to the house. Could you please say why?

[ ]

If Do you agree to participate? is Yes:

Household Demographics

What is the highest level of school the head of the household ever attended?

- ◯ No education
- ◯ Primary
- ◯ Secondary
- ◯ Higher
- ◯ Other (please specify)

[ ]

How many people live in this household?

[ ]

If Do you agree to participate? is Yes:

Demographic and Diarrhea

Now I would like to ask about each household member and if they have had diarrhea in the preceding week. For children (< 9 years), I would like to ask the questions to their caregiver. For adults, I would like to ask the questions to themselves. If any adult is not available and other household member can report accurately, I will record that answer as well. Please start with the youngest household member.

Diarrhea and OCV status

Age	Age (years or months)	Gender	Diarrhea within last 7 days?	Received OCV?
	years / months	Female / Male	Yes / No / Don't know	No / ≤ 6 months / >6 and ≤ 12 months / >12 and ≤ 18 months / >18 and ≤ 24 months / > 24 months
	years / months	Female / Male	Yes / No / Don't know	No / ≤ 6 months / >6 and ≤ 12 months / >12 and ≤ 18 months / >18 and ≤ 24 months / > 24 months
	years / months	Female / Male	Yes / No / Don't know	No / ≤ 6 months / >6 and ≤ 12 months / >12 and ≤ 18 months / >18 and ≤ 24 months / > 24 months
	years / months	Female / Male	Yes / No / Don't know	No / ≤ 6 months / >6 and ≤ 12 months / >12 and ≤ 18 months / >18 and ≤ 24 months / > 24 months
	years / months	Female / Male	Yes / No / Don't know	No / ≤ 6 months / >6 and ≤ 12 months / >12 and ≤ 18 months / >18 and ≤ 24 months / > 24 months

If Diarrhea within last 7 days? is Yes:
Day of diarrhea onset

If Diarrhea within last 7 days? is Yes:
Duration of diarrhea

If Diarrhea within last 7 days? is Yes:
Was the diarrhea severe enough that the member sought medical care?

- ◯ Yes
- ◯ No

If Diarrhea within last 7 days? is Yes and Was the diarrhea severe enough that the member sought medical care? is Yes:

If yes, where did the member go?

If Diarrhea within last 7 days? is Yes and Was the diarrhea severe enough that the member sought medical care? is Yes:

If yes, was this medical setting:

- ◯ Inpatient - hospital, CTC
- ◯ Outpatient - OPD, clinic, ORP
- ◯ Other (please specify) - Pharmacy, Traditional Healer...

Repeat above questions as many times as necessary

If Do you agree to participate? is Yes:

WASH knowledge, Attitude and Practice:

Water

What is the main source of drinking water used by members of your household?

- ◯ Piped into dwelling
- ◯ Piped to yard/ or plot
- ◯ Piped to neighbor
- ◯ Public tap/ standpipe
- ◯ Tube well/ borehole
- ◯ Protected well
- ◯ Unprotected well
- ◯ Protected spring
- ◯ Unprotected spring
- ◯ Rainwater
- ◯ Tanker-truck
- ◯ Cart with small tank
- ◯ Water kiosk
- ◯ Surface water (river, dam, lake, pond, stream, canal, irrigation channel)
- ◯ Bottled water
- ◯ Sachet water
- ◯ Other (please specify)

Do you share the water source with others who are not members of your household?

◯ Yes
◯ No

How long does it take for members of your household to go there, get water, and come back?

Hint: Give total duration in minutes

Do you or your household members do anything to make the water safer to drink?

◯ Boil
◯ Add bleach/ chlorine
◯ Strain it through a cloth
◯ Use water filter
◯ Solar disinfection
◯ Let it stand and settle
◯ No/ nothing
◯ Other (please specify)

Sanitation

What kind of toilet facility do members of your household usually use?

◯ Flush/ pour flush
◯ Pit latrine: pit latrine with slab
◯ Open pit: pit latrine without slab
◯ Composting toilet
◯ Bucket
◯ Hanging toilet
◯ No facility/ bush/ field
◯ Other (please specify)

Observe: Where is that toilet facility located?

◯ In own dwelling
◯ In own yard
◯ Elsewhere

Do you share this facility with others who are not members of your household?

◯ Yes
◯ No

Handwashing

We would like to learn about where members of this household wash their hands. Can you please show me where members of your household most often wash their hands? (Record result and observation.)

Observe: Was water available?

◯ Yes
◯ No

Observe: Was soap available?

◯ Bar Soap
◯ Liquid Soap
◯ Powder soap
◯ No

Observe the type of handwashing facility:

◯ Fixed facility in dwelling (sink/ tap/ tube well)
◯ Fixed facility in yard (sink/ tap/ tube well)
◯ Mobile object observed (bucket/ jug/ kettle)
◯ No handwashing place
◯ Other (please specify)

Can you tell us the critical times of handwashing?
Hint: DO NOT READ ALOUD. Select options that are mentioned.

☐ Before and after preparing food
☐ Before eating food
☐ After using the toilet
☐ After cleaning up a child who used the toilet
☐ Before and after taking care of a sick person
☐ Other (please specify)

If Do you agree to participate? is Yes and In the past month, did your household receive a visit from a CATI team? is Yes:

CATI Activities and Distribution (Skip this section if the household did not receive CATI intervention.)

Did the CATI Team disinfect any part of your household?

◯ Yes
◯ No

If Did the CATI Team disinfect any part of your household? is Yes:
CATI Team disinfected:
Hint: Select all that apply

☐ Disinfected toilet
☐ Disinfected bedding
☐ Disinfected kitchen
☐ Disinfected floor
☐ Disinfected other surface (wall, furniture, utensils)
☐ Don't know

Did the CATI Team test stored drinking water for free chlorine residual (or local term for chlorine testing)?

◯ Yes
◯ No

Did you receive hygiene education related to cholera transmission prevention?

◯ Yes
◯ No

If Did you receive hygiene education related to cholera transmission prevention? is Yes:

How satisfied are you with the training you received on cholera prevention?

◯ Very satisfied
◯ Satisfied
◯ Neither satisfied nor dissatisfied
◯ Dissatisfied
◯ Very dissatisfied.

Can you please tell us the items you received from the CATI Team?

☐ Chlorine tablet or solution to disinfect water
☐ Jerry can or water storage container
☐ Soap and/or laundry powder
☐ ORS
☐ IEC materials
☐ None
☐ Other (please specify)

Did the CATI team administer antibiotic (medicine to prevent or shorten diarrhea) to any member of the household?

◯ Yes
◯ No

If Do you agree to participate? is Yes:

FCR

Could you please provide me with a glass of water that members of your household usually drink?

◯ Yes
◯ No

If Could you please provide me with a glass of water that members of your household usually drink? is Yes:

Observe and record whether the water was collected directly from the source or from a separate storage container.

◯ Direct from Source
◯ Covered Container
◯ Uncovered Container
◯ Unable to Observe

If Could you please provide me with a glass of water that members of your household usually drink? is Yes:

Using the chlorine tester and DPD1, please conduct the FCR test:

Hint: unit is mg/L

Accountability

Are you aware of AAH feedback channels?

◯ Yes
◯ No

Have you shared your complaints/feedback about AAH’s program in your location?

◯ Yes
◯ No

If Have you shared your complaints/feedback about AAH’s program in your location? is Yes:

If yes, which of these have you used in the past to channel your complaint

◯ AAH staff on the ground
◯ Community leaders
◯ Community Volunteers
◯ Toll free line
◯ Other (please specify)

What could be done to make it easier for you to make requests, share concerns, or make a complaint regarding to the cholera intervention

◯ AAH staff on the ground
◯ Community leaders
◯ Suggestion Box
◯ Have community meetings
◯ Toll free line
◯ Dont Know
◯ Other (please specify)

Phase 2B: Follow up CATI Team Form

General

CATI Form number:

Enumerator's Name:

Case Household

Street address:

Community/neighborhood:

District:

CTC Information

CTC Case ID:

Hint: Enter the unique Case ID from line list.

CATI Implementation Information:

ID number of the CATI team that completed the intervention:

Did you survey the Case Household?

◯ Yes
◯ No

If Did you survey the Case Household? is No:

Why did you not survey Case Household?

◯ Was not present at the household
◯ Did not want to participate in the survey
◯ Could not find the household
◯ Other (please specify)

In total, how many neighbors (except the case household) were attempted and surveyed for follow-up? (Complete this question after finishing all follow-up surveys for the CATI.)

Attempted:

Surveyed:

Number of houses attempted but not surveyed [expression]

You reported one or more household(s) could not be surveyed. Please select reasons for each household that could not be surveyed.

Household was not surveyed:

☐ Was not present at the household
☐ Did not want to participate in the survey
☐ Could not find the household
☐ Other (please specify)

Phase 2A Follow up Household Form 9.13.21

Identifier

Unique Household ID: 1117000000000

Follow up CATI Form number:

Please write the "Follow up CATI Form" number here

Enumerator's name

Date

yyyy-mm-dd

Is it a case or neighbor's household?

- ◯ Case
- ◯ Neighbor

Household Information

State:

- ◯ Adamawa
- ◯ Borno
- ◯ Yobe

LGA:

Ward:

Community/neighborhood:

Street address:

GPS

latitude (x.y °)

longitude (x.y °)

altitude (m)

accuracy (m)

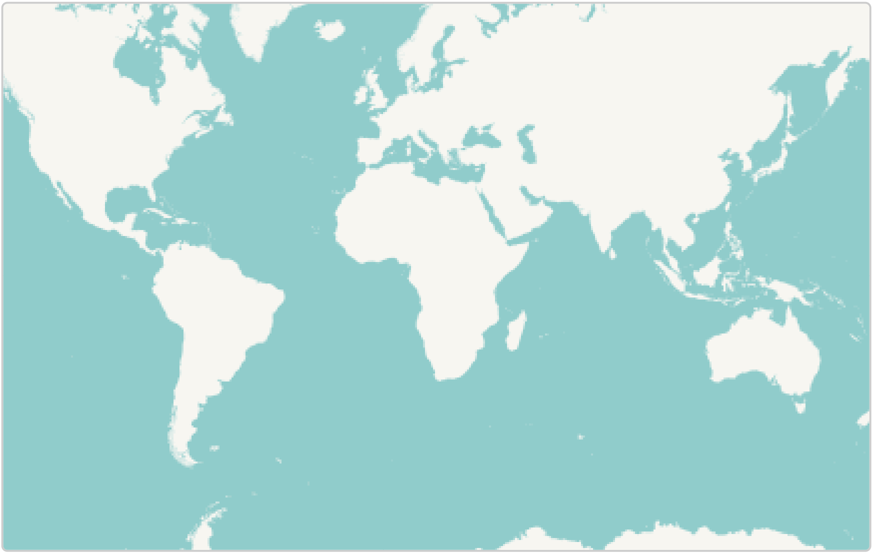

Source: OpenStreetMap (https://www.openstreetmap.org/#map=5/23.93/-102.57)

Consent and respondent's information

WE WILL ONLY SURVEY RESPONDENTS WHO ARE 18 YEARS OR OLDER. Please ask the respondent about their age. If the respondent is younger than 18 years, please request to interview someone from the household who is over 18 years. Please read the follow-up survey consent form to the respondent and obtain oral consent to conduct the survey.

Has the respondent given consent to conduct the survey?

- (●) Yes
- ( ) No
- ( ) No adult at the household

Respondent is:

- ( ) Female
- ( ) Male

Age of the respondent (in years)?

Respondent must be 18 years or older to complete survey.

In the past month, did your household receive a visit from a CATI team?

- (●) Yes
- ( ) No

Why did you not receive CATI intervention

- ◯ The CATI team did not come to the house
- ◯ We did not want the CATI team to come to the house
- ◯ No one was at the house when the CATI team came
- ◯ Do not know
- ◯ Other

You said you did not want the CATI team to come to the house, could you please say why?

Other:

Household Demographics

What is the highest level and grade or year of school the head of the household ever attended?

How many people live in this household?

2

Now I would like to ask about each household member and if they have had diarrhea in the preceding week. For children (< 9 years), I would like to ask the questions to their caregiver. For adults, I would like to ask the questions to themselves. If any adult is not available and other household member can report accurately, I will record that answer as well. Please start with the youngest household member.

Demographic and Diarrhea

1

Member's age:

- ◯ <5 years
- ◯ 5 - 9 years
- ◯ 10 - 19 years
- ◯ 20 - 29 years
- ◯ 30 - 39 years
- ◯ 40 - 49 years
- ◯ 50 - 59 years
- ◯ 60 - 69 years
- ◯ 70- 79 years
- ◯ 80 years and above

* **Member's Sex:**

- ◯ Female
- ◯ Male

In the last seven days, did this member have diarrhea? [Diarrhea is defined as the passage of three or more loose or liquid stools per day]

- ◉ Yes
- ◯ No
- ◯ Don't know
- ◯ Member not available and other household members don't have sufficient knowledge to report diarrhea

* **Day of diarrhea onset:**

yyyy-mm-dd

____

* **Duration of diarrhea:**

number of days

____

Was the diarrhea severe enough that the member sought medical care?

- ◯ Yes
- ◯ No

If yes, where did the member go?

Write name of health facility

____

If yes, where did the member go?

- Inpatient (IPD)
- Outpatient (OPD)
- Unsure

If unsure medical facility was inpatient (IPD) or outpatient (OPD), did respondent stay overnight?

- Yes
- No

Did this member receive any oral cholera vaccine?

- Yes
- No
- Don't know

If yes, approximately when did the member receive the vaccine? (if multiple, record the latest)

- in ≤ six months
- > 6 months and ≤ 12 months
- > 12 months and ≤ 18 months
- > 18 months and ≤ 24 months
- > 24 months

2

Member's age:

- <5 years
- 5 - 9 years
- 10 - 19 years
- 20 - 29 years
- 30 - 39 years
- 40 - 49 years
- 50 - 59 years
- 60 - 69 years
- 70- 79 years
- 80 years and above

*** Member's Sex:**

- ( ) Female
- ( ) Male

In the last seven days, did this member have diarrhea? [Diarrhea is defined as the passage of three or more loose or liquid stools per day]

- ( ) Yes
- (•) No
- ( ) Don't know
- ( ) Member not available and other household members don't have sufficient knowledge to report diarrhea

*** Day of diarrhea onset:**

yyyy-mm-dd

*** Duration of diarrhea:**

number of days

Was the diarrhea severe enough that the member sought medical care?

- ( ) Yes
- ( ) No

If yes, where did the member go?

Write name of health facility

If yes, where did the member go?

- ( ) Inpatient (IPD)
- ( ) Outpatient (OPD)
- ( ) Unsure

If unsure medical facility was inpatient (IPD) or outpatient (OPD), did respondent stay overnight?

- ( ) Yes
- ( ) No

Did this member receive any oral cholera vaccine?

- ( ) Yes
- ( ) No
- ( ) Don't know

If yes, approximately when did the member receive the vaccine? (if multiple, record the latest)

- in ≤ six months
- > 6 months and ≤ 12 months
- > 12 months and ≤ 18 months
- > 18 months and ≤ 24 months
- > 24 months

WASH Knowledge, Attitude and Practice:

» Water

What is the main source of drinking water used by members of your household?

- Piped into dwelling
- Piped to yard / plot
- Piped to neighbor
- Public tap / standpipe
- Tube well / borehole
- Protected well
- Unprotected well
- Protected spring
- Unprotected spring
- Rainwater
- Tanker-truck
- Cart with small tank
- Water kiosk
- Surface water (river, dam, lake, Pond, stream, canal, irrigation channel)
- Bottled water
- Sachet water
- Other (specify)

Other

Do you share the water source with others who are not members of your household?

- Yes
- No

How long does it take for members of your household to go there, get water, and come back? (total duration in minutes)

Minutes

Do you or your household members do anything to make the water safer to drink?

- ◯ Boil
- ◯ Add bleach/chlorine
- ◯ Strain it through a cloth
- ◯ Use water filter
- ◯ Solar disinfection
- ◯ Let it stand and settle
- ◯ No/Nothing
- ◯ Other

Other:

» Sanitation

What kind of toilet facility do members of your household usually use?

- ◯ Flush / pour flush
- ◯ Pit latrine
- ◯ Open pit
- ◯ Composting toilet
- ◯ Bucket
- ◯ Hanging toilet /
- ◯ No facility / bush / field
- ◯ Other

Other

Where is that toilet facility located?

- ◯ In own dwelling
- ◯ In own yard
- ◯ Elsewhere

Do you share this facility with others who are not members of your household?

- Yes
- No

» Handwashing

**We would like to learn about where members of this household wash their hands. Can you please show me where members of your household most often wash their hands? (Record result and observation)**	Yes	No
**Was water available?**	◯	◯
**Was soap available?**	◯	◯

Observe type of handwashing facility.

Please select all types of handwashing facilities available in this household.

- ☐ Fixed facility in dwelling (sink/ tap/ tube well)
- ☐ Fixed facility in yard (sink/ tap/ tube well)
- ☐ Mobile object observed (bucket/ jug/ kettle)
- ☐ No handwashing place
- ☐ Other

Other:

____

**Can you tell us the critical times of handwashing? (Do not read aloud. Select "Yes" for all times mentioned by respondent.)**	Yes	No
**Before and after preparing food**	◯	◯
**Before eating food**	◯	◯
**After using the toilet**	◯	◯
**After cleaning up a child who used the toilet**	◯	◯
**Before and after taking care of a sick person**	◯	◯
**Other**	◯	◯

Other

____

CATI activities and distribution (skip this section if the household did not receive CATI intervention)

Did the CATI team disinfect any part of your household?

- ◯ Yes
- ◯ No

**Disinfected:**	Yes	No
**Disinfected toilet**	◯	◯
**Disinfected bedding**	◯	◯
**Disinfected kitchen**	◯	◯
**Disinfected floor**	◯	◯
**Disinfected other surface (wall, furniture, utensils)**	◯	◯
**Don't know**	◯	◯

Did the CATI team test stored drinking water for free chlorine residual (or local term for chlorine testing)

- ◯ Yes
- ◯ No

Did you receive hygiene education related to cholera transmission prevention?

- ◯ Yes
- ◯ No

**Can you please tell us the items you received from the CATI team?**	Yes	No
**Chlorine tablet or solution to disinfect water**	◯	◯
**Jerry can or water storage container**	◯	◯
**Soap and/or laundry powder**	◯	◯
**ORS**	◯	◯
**IEC materials**	◯	◯
**Other**	◯	◯

Other:

____

Did the CATI team administer antibiotic (medicine to prevent or shorten diarrhea) to any member of the household?

- Yes
- No

FCR

Could you please provide me with a glass of the water that members of your household usually drink?

- Yes
- No

Observe and record whether the water was collected directly from the source or from a separate storage container.

- Direct from Source
- Covered Container
- Uncovered Container
- Unable to Observe

Q27. Using the chlorine tester and DPD1, please conduct the FCR test: (unit: mg/L)

____

Phase 2B Follow up CATI Team Form 9.13.21

General

CATI Form number:

Date

yyyy-mm-dd

Corresponding CTC:

- CTC1
- CTC2
- CTC3
- CTC4
- CTC5
- CTC6
- Other

Other

Write the name of the 'Other' CTC

Enumerator's name:

» Case household

State:

- Adamawa
- Borno
- Yobe

LGA:

Ward:

Community/neighborhood:

Street address:

CTC Information

CTC case ID: (enter the unique case ID from line list)

CTC admission date

yyyy-mm-dd

CATI Implementation Information:

ID number of the CATI team that completed the intervention:

Date CATI was completed

yyyy-mm-dd

Did you survey the case household?

- Yes
- No

Why did you not survey case household?

- Was not present at the household
- Did not want to participate in the survey
- Could not find the household
- Other

Other

In total, how many neighbors (except the case household) were attempted and surveyed for follow-up? (complete this question after finishing all follow-up surveys for the CATI)

Attempted

4

Surveyed

1

You reported one of more household(s) that could not be survey, please select reasons for each household that could not be surveyed

Not surveyed household

1

*** Household was not surveyed**

- ◯ Was not present at the household
- ◯ Did not want to participate in the survey
- ◯ Could not find the household
- ◯ Other

Other

2

*** Household was not surveyed**

- ◯ Was not present at the household
- ◯ Did not want to participate in the survey
- ◯ Could not find the household
- ◯ Other

Other

3

*** Household was not surveyed**

- ◯ Was not present at the household
- ◯ Did not want to participate in the survey
- ◯ Could not find the household
- ◯ Other

Other
