# Supplementary material for: Case-area targeted interventions and free chlorine residual in household drinking water: An observational cohort study during a cholera outbreak in Northeast Nigeria
Source: PLoS Negl Trop Dis. 2025 Jan 27;19(1):e0012731. doi: 10.1371/journal.pntd.0012731 (PMC11771888; doi:10.1371/journal.pntd.0012731)
Supplement: S2 Appendix — (PDF) [file pntd.0012731.s002.pdf]

S2 Appendix: Descriptive Statistics, Yobe State

Table 1. Respondent, Household, and CATI Characteristics, Yobe State

	Case Households	Neighbor Households
**CATI Response** (% (n))		
Received CATI (Phase 1)	360	12,390
Revisited for Phase 2 Follow-Up	71.1 (256)	66.4 (8,225)
**Household Characteristics** (% (n))		
Household Size [a]	6 (5, 9)	5 (3, 7)
Respondent Age [a]	32 (25, 40)	28 (22, 37)
Respondent Female [b]	70.3 (180)	76.5 (6,295)
Highest Household Education Level [b]		
None	15.6 (40)	6.4 (529)
Islamic	37.1 (95)	39.9 (3,277)
Primary	3.5 (9)	4.3 (351)
Secondary	14.8 (38)	27.2 (2,231)
Post-secondary	28.9 (74)	22.2 (1,821)
**Household WASH and Environmental Characteristics** [c] **– Phase 2** (% [n])		
Water Source [d]		
Protected	37.7 (92)	20.7 (1,572)
Purchased	52.5 (128)	72.7 (5,530)
Piped	4.1 (10)	5.3 (401)
Unimproved	5.7 (14)	1.4 (103)
Distance to CTC [a]	4.3 (2.3, 7.5)	-
Time to Water Source in Minutes [a]	10 (5, 15)	15 (10, 20)
Water Treatment [b]		
None	10.0 (25)	34.2 (2,733)
Add Bleach/Chlorine	84.4 (211)	56.9 (4,545)
Let Stand and Settle	3.6 (9)	1.3 (102)
Strain Through Cloth	1.2 (3)	6.1 (487)
Other	0.8 (2)	1.5 (119)
Shared Latrine [b, e]	68.0 (174)	86.4 (7,106)
Improved Latrine [b, f]	29.7 (76)	35.7 (2,937)
Handwashing Availability [b]		

Basic (Soap +/- Water)	84.7 (216)	76.6 (6,265)
Limited (Water)	10.2 (26)	20.9 (1,712)
None	5.1 (13)	2.5 (204)
Knowledge of Key Handwash Times [b]		
Before Food Preparation	96.9 (248)	74.0 (6,083)
Before Eating	99.2 (254)	99.3 (8,164)
After Using Toilet	97.3 (249)	99.2 (8,163)
Before Cleaning a Child	97.3 (249)	96.2 (7,910)
When Caring for a Sick Person	77.3 (198)	45.7 (3,760)

[a] (Median (IQR))

[b] (% (n))

[c] Most humanitarian organizations like SI and ACF use humanitarian WASH standards, such as the Sphere Standards (1) or UNHCR for WASH Access (2), rather than Sustainable Development Goal standards (3). Thus, the terms improved and unimproved are used herein.

[d] Protected water source: tube well/borehole, rainwater, public tap/standpipe, protected well, protected spring. Piped: piped into dwelling, piped to neighbor, piped to yard/plot. Purchased: bottled, cart with small tank, water kiosk, water sachet. Unimproved: tanker truck, unprotected well, surface water.

[e] Shared latrine: Household shares latrine with at least one other household.

[f] Improved latrine: flush, pour flush, pit latrine (with slab), composting toilet.

CATI: case-area targeted intervention. CTC: cholera treatment center. IQR: interquartile range. n: number. UNHCR: United Nations High Commissioner for Refugees. WASH: water, sanitation, and hygiene.

Table 2. Reported CATI Activities by Phase, Yobe State

	Household-Report (Phase 2)	Team-Report (Phase 1)	p-value
**Cases** (n)	256	331	
Supplies (% (n))			
Aquatabs	95.7 (245)	98.8 (327)	0.019
Soap	92.6 (237)	81.3 (269)	<0.001
Jerry Can	87.5 (224)	90.0 (298)	0.28
Complete Supplies[1]	86.3 (221)	74.9 (248)	<0.001
Activities (% (n))			
Hygiene Promotion	99.2 (254)	99.4 (329)	0.58
Latrine Disinfection	98.4 (252)	99.7 (330)	0.036
Bedding Disinfection	94.1 (241)	95.5 (315)	0.47
Complete Activities[2]	92.6 (237)	95.2 (314)	0.19
Complete CATI	81.3 (208)	70.1 (232)	0.003
**Neighbors** (n)	8,225	10,329	
Supplies (% (n))			
Aquatabs	47.5 (3,903)	80.4 (8,297)	<0.001
Soap	45.7 (3,757)	36.4 (3,756)	<0.001
Complete Supplies[1]	39.0 (3,210)	33.1 (3,414)	<0.001
Activities (% (n))			
Hygiene Promotion	98.9 (8,135)	99.3 (10,256)	<0.001
Latrine Disinfection	98.2 (8,074)	99.4 (10,270)	<0.001
Bedding Disinfection	90.3 (7,427)	84.2 (8,697)	<0.001
Complete Activities[2]	89.2 (7,330)	83.5 (8,623)	<0.001
Complete CATI	37.4 (3,075)	32.5 (3,359)	<0.001

[1] Complete Supplies means the household received all of the following supplies: Aquatabs, soap, IEC materials, and (if a case household) a jerry can.

[2] Complete Activities means the household received all of the following activities: hygiene promotion, latrine disinfection, and bedding disinfection.

CATI: case-area targeted intervention. IEC: information, education, and communication. n: number.

References

(1) The Sphere Project. The Sphere Handbook, Ch. 6 Water Supply, Sanitation, and Hygiene Promotion. https://handbook.spherestandards.org/en/sphere/#ch006; 2018.

(2) UNHCR. Emergency Handbook, WASH in Emergencies. https://emergency.unhcr.org/emergency-assistance/water-sanitation-and-hygiene/wash-emergencies; 2024.

(3) WHO/UNICEF Joint Monitoring Programme for Water and Sanitation. WASH in the 2030 Agenda. 2017.
