# Supplementary material for: Case-area targeted interventions and free chlorine residual in household drinking water: An observational cohort study during a cholera outbreak in Northeast Nigeria
Source: PLoS Negl Trop Dis. 2025 Jan 27;19(1):e0012731. doi: 10.1371/journal.pntd.0012731 (PMC11771888; doi:10.1371/journal.pntd.0012731)
Supplement: S3 Appendix — (PDF) [file pntd.0012731.s003.pdf]

S3 Appendix: Sensitivity Analyses

Methods

During analysis, missing data matrices revealed a pattern indicating missingness at random (MAR) in our dataset. We thus conducted sensitivity analyses comparing a complete case (CC) analysis with four imputed data sets. Missing values were imputed using predictive mean matching (PMM) with three distinct correlations (0.3, 0.2, and 0.1) as well as a random forest (RF) imputation model with a correlation of 0.1. The random forest imputation model was used in the final results due to its ability to handle interactions between variables, manage missing data patterns with some degree of randomness, such as MAR, deal with both continuous and categorical variables and express increased robustness against outliers.

Results

Complete case (CC) data included 4,243 observations in Adamawa and 3,749 in Borno. Imputed data sets included 4,997 observations in Adamawa and 4,040 in Borno. In both Adamawa and Borno water source and type of handwashing station were the only variables missing data. In Adamawa, data on water source was missing in 15.0% of observations and data on handwashing station in <1.0% of observations. In Borno, data on water source was missing in 5.0% of observations and data on handwashing station in <1% of cases.

In univariate sensitivity analyses, certain associations which were significant in the complete case model lost significance in imputed models. In Adamawa, households that received hygiene promotion had a significant increased odds of low FCR than those that did not in the CC analysis (aOR 5.43 [95%CI 3.00, 10.1]; p<0.001), while this association was lost in the imputed data sets. Similarly, in the CC analysis, using piped water compared to a protected water source and sharing a latrine increased odds of low FCR (aOR 2.16 [95%CI 1.58, 3.03]; p<0.001 and aOR 1.92 [95%CI 1.64, 2.24]; p<0.001 respectively). Increasing distance to the CTC showed a decreased odds of low FCR (aOR 0.96 [95%CI 0.94, 0.98]; p<0.001). These associations likewise lost significance in the imputed datasets. Conversely, in Adamawa, the imputed data sets showed that use of purchased water over a protected water source decreased the odds of low FCR, acting as a protective factor (aOR 0.54 [95%CI 0.44, 0.67]; p<0.001 in the RF data set), an association not seen in the CC model. Significance of other associations remained the same between CC and imputed datasets. In Borno univariate sensitivity analysis, the only variable that changed association was latrine sharing, which increased the odds of low FCR in the CC analysis (aOR 2.71 [95%CI 1.95, 3.73]; p<0.001) and did not have a significant association in the imputed data analyses. In Borno, significance of all other associations remained the same between CC and imputed analyses. However, results were consistently attenuated in imputed analyses.

In multivariate sensitivity analyses, associations stayed consistent between CC and imputed data sets, though the strength varied between data sets. In Adamawa, imputed data sets showed a higher odds ratio of low FCR in households with a handwash station with only water than the CC data set (aOR 2.65 [95%CI 2.00, 3.50]; p<0.001 in the RF data set vs aOR 2.40 [95%CI 1.88, 3.07]; p<0.001 in CC data). The odds ratio of households with no handwashing station available was also higher for low FCR concentrations in imputed data sets compared to the CC data (aOR 2.53 [95%CI 1.84, 3.47]; p<0.001 in the RF data set vs aOR 2.22 [95%CI 1.69, 2.93]; p<0.001 in CC data). In Borno, imputed data sets showed

use of purchased water had a slightly lower increased odds of inadequate FCR concentrations (aOR 4.59 [95%CI 1.61, 13.1]; $p=0.004$ in the RF data set vs aOR 5.11 [95%CI 1.69, 15.5]; $p=0.004$ in CC data) as did ring coverage of 10-25% (aOR 3.63 [95%CI 1.16, 11.4]; $p=0.027$ in the RF data set vs aOR 3.44 [95%CI 1.10, 10.8]; $p=0.034$ in CC data) and absence of handwashing station (aOR 6.02 [95%CI 2.65, 13.7]; $p<0.001$ in the RF data set vs aOR 5.78 [95%CI 2.53, 13.2]; $p<0.001$ in CC data).

Adamawa FCR Multivariate Sensitivity Analysis															
	Complete Data			Imputed Data, Corr 0.3			Imputed Data, Corr 0.2			Imputed Data, Corr 0.1			Imputed Data, Corr 0.1 - Random Forest		
**Characteristic**	**aOR**[1]	**95% CI**[1]	**p-value**	**aOR**[1]	**95% CI**[1]	**p-value**	**aOR**[1]	**95% CI**[1]	**p-value**	**aOR**[1]	**95% CI**[1]	**p-value**	**aOR**[1]	**95% CI**[1]	**p-value**
**Aquatabs**	1.08	0.92, 1.27	0.3	1.06	0.90, 1.24	0.5	1.06	0.90, 1.24	0.5	1.07	0.91, 1.26	0.4	1.06	0.90, 1.24	0.5
**Water Source**															
Protected	—	—		—	—		—	—		—	—		—	—	
Piped	1.14	1.04, 1.25	**0.006**	1.12	1.03, 1.21	**0.007**	1.12	1.03, 1.21	**0.008**	1.11	1.02, 1.21	**0.014**	1.11	1.04, 1.19	0.002
Purchased	0.68	0.55, 0.83	**<0.001**	0.68	0.55, 0.83	**<0.001**	0.68	0.55, 0.83	**<0.001**	0.68	0.56, 0.83	**<0.001**	0.67	0.55, 0.82	<0.001
Unimproved	0.81	0.59, 1.09	0.2	0.79	0.58, 1.09	0.2	0.79	0.58, 1.10	0.2	0.82	0.61, 1.09	0.2	0.80	0.59, 1.09	0.2
**Hygiene Promotion**	1.24	0.96, 1.62	0.11	1.23	0.98, 1.56	0.076	1.23	0.98, 1.56	0.078	1.23	0.98, 1.56	0.078	1.24	0.98, 1.56	0.069
**Ring Coverage**															
0-10%	—	—		—	—		—	—		—	—		—	—	
>10-25%	0.84	0.48, 1.46	0.5	0.81	0.47, 1.41	0.5	0.80	0.46, 1.39	0.4	0.83	0.47, 1.45	0.5	0.82	0.47, 1.42	0.5
>25%	0.68	0.35, 1.31	0.3	0.68	0.34, 1.38	0.3	0.68	0.33, 1.39	0.3	0.73	0.35, 1.53	0.4	0.75	0.36, 1.52	0.4
**Handwash Station**															
Soap (+/-Water)	—	—		—	—		—	—		—	—		—	—	
Water	2.39	1.87, 3.05	**<0.001**	2.65	1.96, 3.56	**<0.001**	2.72	2.01, 3.69	**<0.001**	2.78	2.09, 3.70	**<0.001**	2.66	2.01, 3.52	<0.001
Not available	2.22	1.69, 2.93	**<0.001**	2.55	1.78, 3.66	**<0.001**	2.63	1.78, 3.90	**<0.001**	2.75	1.94, 3.90	**<0.001**	2.57	1.87, 3.51	<0.001
**Distance to CTC**	1.02	0.95, 1.11	0.6	1.00	0.93, 1.08	>0.9	1.00	0.93, 1.08	>0.9	0.99	0.92, 1.07	0.8	0.99	0.92, 1.07	0.8
**Aquatabs * Water Source**															
*Aquatabs * Piped*	0.89	0.60, 1.32	0.6	0.90	0.64, 1.26	0.5	0.90	0.65, 1.27	0.6	0.90	0.62, 1.30	0.6	0.89	0.66, 1.20	0.4
*Aquatabs * Purchased*	0.40	0.21, 0.76	**0.005**	0.44	0.24, 0.80	**0.007**	0.44	0.24, 0.79	**0.006**	0.43	0.24, 0.77	**0.004**	0.44	0.25, 0.78	0.004
*Aquatabs * Unimproved*	0.10	0.01, 1.10	0.060	0.11	0.01, 1.14	0.064	0.10	0.01, 1.06	0.056	0.13	0.01, 1.38	0.091	0.12	0.01, 1.19	0.070

[1] OR = Odds Ratio, CI = Confidence Interval

Borno FCR Multivariate Sensitivity Analysis

	Complete Data			Imputed Data, Corr 0.3			Imputed Data, Corr 0.2			Imputed Data, Corr 0.1			Imputed Data, Corr 0.1 - Random Forest		
**Characteristic**	**aOR**[1]	**95% CI**[1]	**p-value**	**aOR**[1]	**95% CI**[1]	**p-value**	**aOR**[1]	**95% CI**[1]	**p-value**	**aOR**[1]	**95% CI**[1]	**p-value**	**aOR**[1]	**95% CI**[1]	**p-value**
**Aquatabs**	0.12	0.05, 0.27	**<0.001**	0.12	0.05, 0.26	**<0.001**	0.12	0.05, 0.26	**<0.001**	0.12	0.05, 0.26	**<0.001**	0.12	0.05, 0.25	<0.001
**Water Source**															
Protected	—	—		—	—		—	—		—	—		—	—	
Piped	0.29	0.05, 1.70	0.2	0.42	0.07, 2.55	0.3	0.43	0.07, 2.64	0.4	0.44	0.07, 2.59	0.4	0.42	0.07, 2.52	0.3
Purchased	5.03	1.65, 15.3	**0.004**	4.63	1.66, 12.9	**0.003**	4.37	1.41, 13.5	**0.011**	4.50	1.61, 12.6	**0.004**	4.45	1.57, 12.7	0.005
**Hygiene Promotion**	1.02	0.58, 1.79	>0.9	1.11	0.66, 1.88	0.7	1.11	0.66, 1.87	0.7	1.11	0.66, 1.88	0.7	1.11	0.65, 1.87	0.7
**Ring Coverage**															
0-10%	—	—		—	—		—	—		—	—		—	—	
>10-25%	3.47	1.11, 10.8	**0.032**	3.70	1.18, 11.6	**0.025**	3.69	1.18, 11.6	**0.025**	3.69	1.18, 11.6	**0.025**	3.69	1.18, 11.6	0.025
>25%	1.32	0.31, 5.57	0.7	1.36	0.30, 6.08	0.7	1.36	0.29, 6.47	0.7	1.34	0.30, 5.97	0.7	1.30	0.33, 5.16	0.7
**Handwash Station**															
Soap (+/-Water)	—	—		—	—		—	—		—	—		—	—	
Water	2.60	1.62, 4.16	**<0.001**	2.55	1.63, 3.99	**<0.001**	2.56	1.64, 4.00	**<0.001**	2.55	1.63, 3.99	**<0.001**	2.56	1.64, 4.01	<0.001
Not available	5.77	2.52, 13.2	**<0.001**	5.91	2.56, 13.6	**<0.001**	6.11	2.70, 13.9	**<0.001**	6.10	2.70, 13.8	**<0.001**	6.02	2.64, 13.7	<0.001
**Distance to CTC**	0.86	0.80, 0.94	**<0.001**	0.90	0.83, 0.97	**0.007**	0.90	0.83, 0.97	**0.007**	0.90	0.83, 0.97	**0.007**	0.90	0.83, 0.97	0.006

[1] OR = Odds Ratio, CI = Confidence Interval
