# Supplementary material for: Case-area targeted interventions and free chlorine residual in household drinking water: An observational cohort study during a cholera outbreak in Northeast Nigeria
Source: PLoS Negl Trop Dis. 2025 Jan 27;19(1):e0012731. doi: 10.1371/journal.pntd.0012731 (PMC11771888; doi:10.1371/journal.pntd.0012731)
Supplement: S4 Appendix — (PDF) [file pntd.0012731.s004.pdf]

S4 Appendix. CATI Phase 2 Diagnostic and Model Fit Analyses

Adamawa

Variance Inflation Factor (VIF)

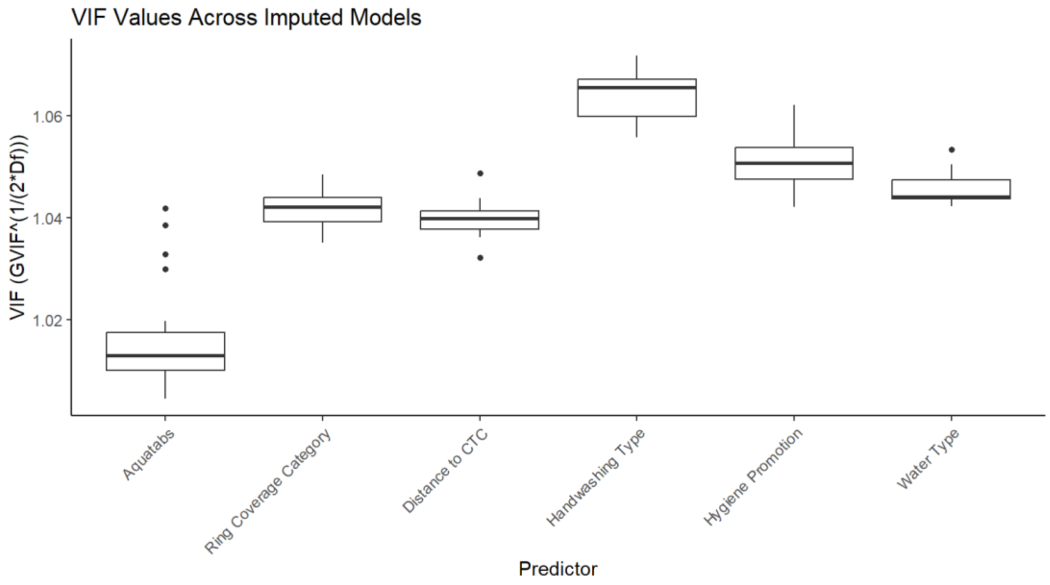

Comments:

All predictors exhibit VIF values close to or below 1.06, which is well within the commonly accepted thresholds (VIF < 5 or 10) indicating little to no multicollinearity. The consistent and low VIF values suggest that the model is stable and free from significant multicollinearity issues, allowing for reliable interpretation of the coefficients.

Univariate Analyses

Adamawa FCR Univariate Sensitivity Analysis

	Complete Case			Impute Corr 0.3			Impute Corr 0.2			Impute Corr 0.1			Impute Corr 0.1 - RF		
**Characteristic**	**OR**[1]	**95% CI**[1]	**p-value**	**OR**[1]	**95% CI**[1]	**p-value**	**OR**[1]	**95% CI**[1]	**p-value**	**OR**[1]	**95% CI**[1]	**p-value**	**OR**[1]	**95% CI**[1]	**p-value**
**Aquatabs**	0.92	0.78, 1.08	0.29	0.96	0.83, 1.11	0.6	0.96	0.83, 1.11	0.6	0.96	0.83, 1.11	0.6	0.96	0.83, 1.11	0.6
**Hygiene Promotion**	5.43	3.00, 10.1	**<0.001**	1.22	0.95, 1.58	0.12	1.22	0.95, 1.58	0.12	1.22	0.95, 1.58	0.12	1.22	0.95, 1.58	0.12
**Water Type**															
Protected	—	—		—	—		—	—		—	—		—	—	
Piped	2.16	1.58, 3.03	**<0.001**	1.07	0.96, 1.19	0.2	1.07	0.96, 1.19	0.2	1.06	0.94, 1.18	0.3	1.06	0.97, 1.16	0.2
Purchased	0.90	0.71, 1.16	0.42	0.54	0.42, 0.70	**<0.001**	0.55	0.43, 0.70	**<0.001**	0.55	0.45, 0.69	**<0.001**	0.54	0.44, 0.67	**<0.001**
Unimproved	0.62	0.29, 1.45	0.24	0.47	0.21, 1.08	0.075	0.47	0.21, 1.08	0.076	0.53	0.25, 1.11	0.091	0.50	0.23, 1.08	0.079
**Handwash Type**															
Soap (+/-Water)	—	—		—	—		—	—		—	—		—	—	
Water	12.9	9.52, 17.8	**<0.001**	4.09	3.03, 5.50	**<0.001**	4.19	3.11, 5.65	**<0.001**	4.24	3.14, 5.72	**<0.001**	4.11	3.07, 5.50	**<0.001**
Not available	24.6	14.8, 45.2	**<0.001**	3.56	2.49, 5.09	**<0.001**	3.65	2.54, 5.24	**<0.001**	3.72	2.57, 5.37	**<0.001**	3.58	2.51, 5.09	**<0.001**
**Distance to CTC**	0.96	0.94, 0.98	**<0.001**	0.96	0.90, 1.04	0.3	0.96	0.90, 1.04	0.3	0.96	0.90, 1.04	0.3	0.96	0.90, 1.04	0.3
**CATI Ring Coverage**															
0-10%	—	—		—	—		—	—		—	—		—	—	
>10-25%	0.86	0.72, 1.01	0.072	0.80	0.48, 1.34	0.4	0.80	0.48, 1.34	0.4	0.80	0.48, 1.34	0.4	0.80	0.48, 1.34	0.4
>25%	0.97	0.79, 1.20	0.79	0.74	0.38, 1.43	0.4	0.74	0.38, 1.43	0.4	0.74	0.38, 1.43	0.4	0.74	0.38, 1.43	0.4

[1] OR = Odds Ratio, CI = Confidence Interval

Comments:

Aquatabs were included in the model due to their logical connection to increasing FCR levels in households, aligning with the theory of change that providing water treatment supplies directly impacts household water quality. Ring coverage was also included based on research highlighting the influence of social networks on household behaviors, as individual behaviors do not occur in isolation. The proportion of households in a ring receiving water treatment supplies and education is theorized to influence community-level behavior, which in turn may affect individual household practices.

In Borno, Aquatabs distribution and distance to CTC significantly contributed to the model in both univariate analysis and ANOVA Wald testing, while ring coverage demonstrated contributions in univariate analysis and borderline significance in ANOVA Wald testing. Given that the theory of change applies consistently across states, and the model aims to test across diverse settings, we standardized the model framework to ensure comparability between states.

Mean Residuals by Grouping Factor (CATI Ring)

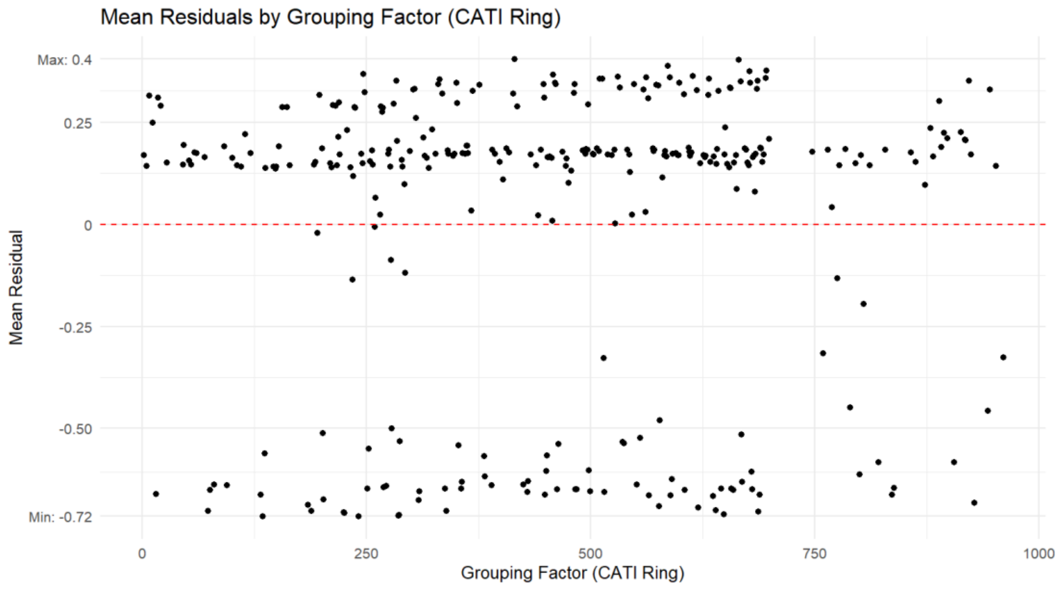

Comments:

The model demonstrates systematic overprediction for groups where the vast majority of observations (97%) have protective FCR levels ≥0.2 mg/L, suggesting a bias in classifying conditions as unsafe when they are actually safe. This overprediction may stem from unmeasured factors or data quality limitations for households with protective FCR levels. Despite this limitation, the model provides a reasonable fit for predicting unsafe conditions (low FCR), where residuals largely fall within acceptable bounds. This ensures that necessary interventions are prioritized for high-risk groups. While the model effectively identifies unsafe outcomes, stakeholders should interpret predictions cautiously for groups with predominantly protective FCR levels to avoid unnecessary interventions and resource allocation.

Summary of ANOVA Wald Test Results Across Imputations

Mean statistics and variability across imputations

Predictor	Mean DF	Mean Chi-Square	Mean P-Value	SD of P-Value
hwash_type	2.000	<0.001	<0.001	<0.001
wat_type	3.000	<0.001	<0.001	<0.001
aquatab:wat_type	3.000	11.828	0.008	0.002
hyg_prom	1.000	2.976	0.086	0.016
aquatab	1.000	<0.001	<0.001	<0.001
ctc_dist	1.000	0.244	0.626	0.055
cov_cat	2.000	0.203	0.904	0.017

Results are based on Wald test from GEE model across multiple imputations.

Borno

Variance Inflation Factor (VIF)

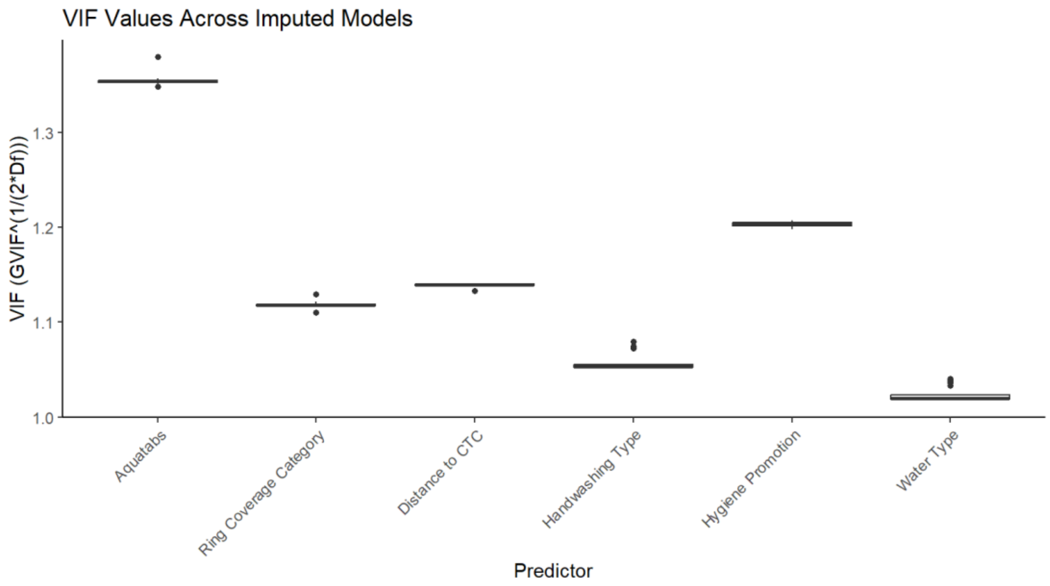

Comments:

All predictors exhibit VIF values close to or below 1.06, which is well within the commonly accepted thresholds (VIF < 5 or 10) indicating little to no multicollinearity. The consistent and low VIF values suggest that the model is stable and free from significant multicollinearity issues, allowing for reliable interpretation of the coefficients.

Univariate Analyses

Borno FCR Univariate Sensitivity Analysis

	Complete Case			Impute Corr 0.3			Impute Corr 0.2			Impute Corr 0.1			Impute Corr 0.1 - RF		
**Characteristic**	**OR**[1]	**95% CI**[1]	**p-value**	**OR**[1]	**95% CI**[1]	**p-value**	**OR**[1]	**95% CI**[1]	**p-value**	**OR**[1]	**95% CI**[1]	**p-value**	**OR**[1]	**95% CI**[1]	**p-value**
**Aquatabs**	0.05	0.03, 0.08	**<0.001**	0.10	0.05, 0.18	**<0.001**	0.10	0.05, 0.18	**<0.001**	0.10	0.05, 0.18	**<0.001**	0.10	0.05, 0.18	**<0.001**
**Hygiene Promotion**	4.00	2.76, 5.70	**<0.001**	1.79	1.07, 2.99	**0.026**	1.79	1.07, 2.99	**0.026**	1.79	1.07, 2.99	**0.026**	1.79	1.07, 2.99	**0.026**
**Water Type**															
Protected	—	—		—	—		—	—		—	—		—	—	
Piped	0.68	0.30, 1.97	0.42	0.86	0.27, 2.70	0.8	0.87	0.28, 2.73	0.8	0.88	0.29, 2.72	0.8	0.87	0.28, 2.71	0.8
Purchased	10.1	2.25, 178	**0.021**	2.85	1.26, 6.46	**0.012**	2.74	1.11, 6.79	**0.030**	2.82	1.25, 6.35	**0.012**	2.72	1.22, 6.07	**0.014**
**Handwash Type**															
Soap (+/-Water)	—	—		—	—		—	—		—	—		—	—	
Water	7.07	5.14, 9.82	**<0.001**	4.23	2.78, 6.44	**<0.001**	4.23	2.78, 6.44	**<0.001**	4.24	2.78, 6.46	**<0.001**	4.23	2.78, 6.44	**<0.001**
Not available	35.1	11.1, 214	**<0.001**	9.83	5.06, 19.1	**<0.001**	10.1	5.28, 19.2	**<0.001**	10.1	5.31, 19.3	**<0.001**	9.94	5.17, 19.1	**<0.001**
**Distance to CTC**	0.88	0.85, 0.92	**<0.001**	0.90	0.84, 0.96	**0.001**	0.90	0.84, 0.96	**0.001**	0.90	0.84, 0.96	**0.001**	0.90	0.84, 0.96	**0.001**
**CATI Ring Coverage**															
0-10%	—	—		—	—		—	—		—	—		—	—	
>10-25%	16.8	7.64, 47.3	**<0.001**	16.5	5.85, 46.6	**<0.001**	16.5	5.85, 46.6	**<0.001**	16.5	5.85, 46.6	**<0.001**	16.5	5.85, 46.6	**<0.001**
>25%	10.4	3.95, 42.4	**<0.001**	8.84	3.26, 24.0	**<0.001**	8.84	3.26, 24.0	**<0.001**	8.84	3.26, 24.0	**<0.001**	8.84	3.26, 24.0	**<0.001**

[1] OR = Odds Ratio, CI = Confidence Interval

Comments:

Ring coverage was also included based on research highlighting the influence of social networks on household behaviors, as individual behaviors do not occur in isolation. The proportion of households in a ring receiving water treatment supplies and education is theorized to influence community-level behavior, which in turn may affect individual household practices. Ring coverage additionally was associated with changes in FCR levels in univariate analysis. All other covariates showed significant associations in both univariate analyses and ANOVA Wald Testing.

Mean Residuals by Grouping Factor (CATI Ring)

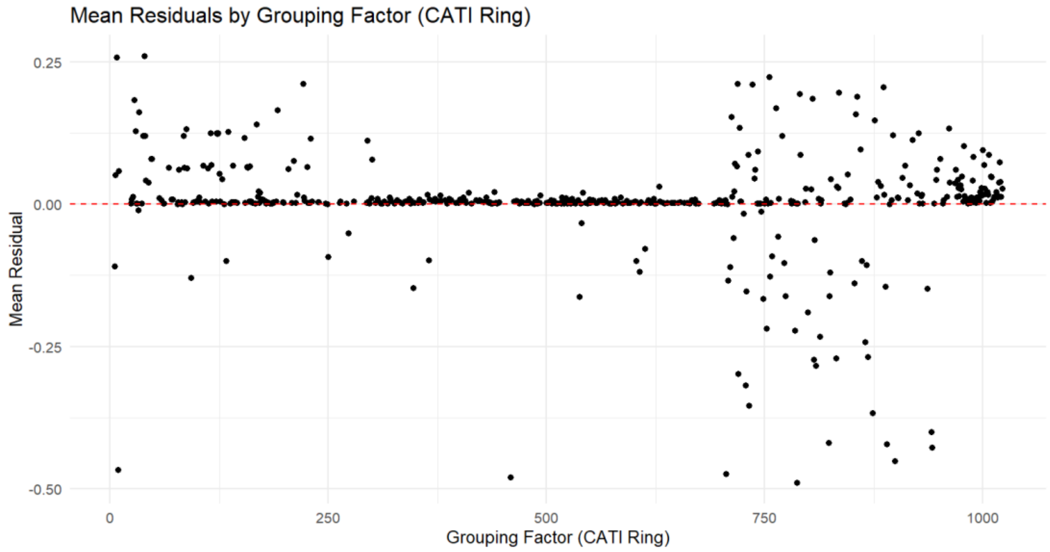

Comments:

The presented model demonstrates a reasonable fit, with most residuals distributed near zero and within an acceptable range. The model demonstrates systematic overprediction for groups where the vast majority of observations (97%) have protective FCR levels ≥0.2 mg/L, suggesting a bias in classifying conditions as unsafe when they are actually safe. This overprediction may stem from unmeasured factors or data quality limitations for households with protective FCR levels. Despite this limitation, the model provides a good fit for predicting unsafe conditions (low FCR), where residuals largely fall within acceptable bounds. This ensures that necessary interventions are prioritized for high-risk groups. While the model effectively identifies unsafe outcomes, stakeholders should interpret predictions cautiously for groups with predominantly protective FCR levels to avoid unnecessary interventions and resource allocation.

Summary of ANOVA Wald Test Results Across Imputations

Mean statistics and variability across imputations

Predictor	Mean DF	Mean Chi-Square	Mean P-Value	SD of P-Value
aquatab	1.000	<0.001	<0.001	<0.001
hwash_type	2.000	<0.001	<0.001	<0.001
hyg_prom	1.000	<0.001	<0.001	<0.001
ctc_dist	1.000	<0.001	<0.001	<0.001
wat_type	2.000	9.480	0.009	0.002
cov_cat	2.000	4.893	0.087	0.005

Results are based on Wald test from GEE model across multiple imputations.
